# Etiology of *TP53* mutated complex karyotype acute myeloid leukemia

**DOI:** 10.1038/s41375-025-02835-9

**Published:** 2025-12-19

**Authors:** Anna Fedenko, Honorata Czapinska, Alwin Krämer, Friedrich Stölzel, Tilmann Bochtler, Matthias Bochtler

**Affiliations:** 1 International Institute of Molecular and Cell Biology, Warsaw, Poland; 2 Clinical Cooperation Unit Molecular Hematology/Oncology, German Cancer Research Center (DKFZ), Heidelberg, Germany; 3 Department of Internal Medicine V, University of Heidelberg, Heidelberg, Germany; 4 Department of Hematology and Oncology, Universitätsklinikum Carl Gustav Carus an der Technischen Universität Dresden, Dresden, Germany; 5 Division of Stem Cell Transplantation and Cellular Immunotherapy, University Hospital Schleswig-Holstein, Campus Kiel, Kiel, Germany; 6 Department of Medical Oncology, National Center for Tumor Diseases, Heidelberg University Hospital, Heidelberg, Germany; 7 Institute of Biochemistry and Biophysics PAS, Warsaw, Poland

**Keywords:** Acute myeloid leukaemia

## Abstract

**Figure:**
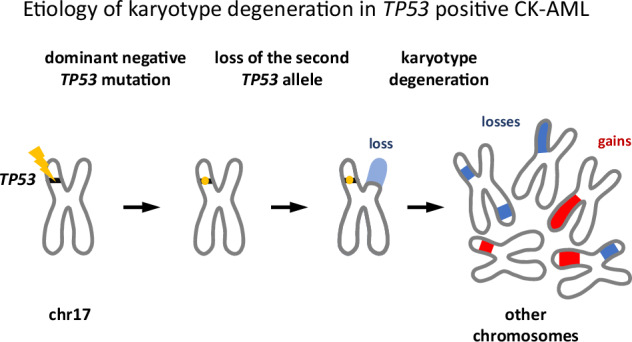
Schematic view of the development of CK-AML driven by the *TP53* absence. The occurrence of the first, often dominant negative *TP53* mutation is quickly followed by the loss of the second *TP53* allele and numerous further chromosomal aberrations.

The occurrence of the first, often dominant negative *TP53* mutation is quickly followed by the loss of the second *TP53* allele and numerous further chromosomal aberrations.

## To the Editor

Complex karyotype acute myeloid leukemia (CK-AML) is defined by three or more unrelated chromosomal aberrations without prognostically favorable inversions and balanced rearrangements. It accounts for 10–14% of AML cases and is associated with an adverse genetic risk [1], poor prognosis [2], and resistance to conventional chemotherapeutic agents [3, 4]. More than half of all CK-AML patients have somatic *TP53* mutations [5–7], which are linked to distinct mutational and cytogenetic profiles and confer a particularly bad prognosis [2, 3]. *TP53* mutation-positive CK-AML is increasingly recognized as a distinct disease, but its pathophysiology remains poorly understood. In particular, the temporal order of mutations and karyotype aberrations is uncertain, since these events typically precede diagnosis and detailed molecular characterization. In this study, we apply a simple mathematical model to infer the proportion of cells that carry *TP53*-related mutational and karyotypic alterations based on copy number variation (CNV) and variant allele frequency (VAF). We show that *TP53* mutations do not consistently precede other driver mutations. However, in CK-AML patients, they are typically dominant negative, and rapidly followed by the loss of the other *TP53* allele and overall karyotype degeneration.

We performed WES and CNV sequencing of 2 normal karyotype (NK) and 33 CK-AML patients: 17 *TP53* mutation-positive (*TP53*_mut_) and 16 *TP53* mutation-negative (*TP53*_wt_) (Supplementary Methods, Tables S1–S3, Fig. S1–S3). As a criterion for CK-AML, we required at least three chromosome abnormalities. None of the patients had t(8;21)(q22;q22) (RUNX1::RUNX1T1), inv(16)(p13.1q22) (CBFB::MYH11), t(16;16)(p13.1;q22) (CBFB::MYH11) or t(15;17)(q22;q12) (PML::RARA) rearrangements, which confer a favorable clinical outcome and are thus exempted from the CK-AML definition [8]. 22 CK-AML samples were collected without bias. 11 samples were from an earlier chromothripsis project [9] and, therefore, were selected for the presence of marker chromosomes. The *TP53*_mut_ cohort included 13 treatment-naïve and 4 pretreated cases (after chemo- or radiation therapy including one case of MDS secondary to chemotherapy for hairy cell leukemia). *TP53*_wt_ patients were all treatment-naïve. In agreement with previous reports [10, 11], overall survival was significantly better for the *TP53*_wt_ group (Fig. S4A), and the difference could not be attributed to different treatment regimens (Table S2). Among the *TP53*_mut_ patients, high VAF values were negatively associated with OS (Pearson r = −0.85, *p* = 3 × 10^−^^5^) (Fig. S4BC).

**Fig. 1:**
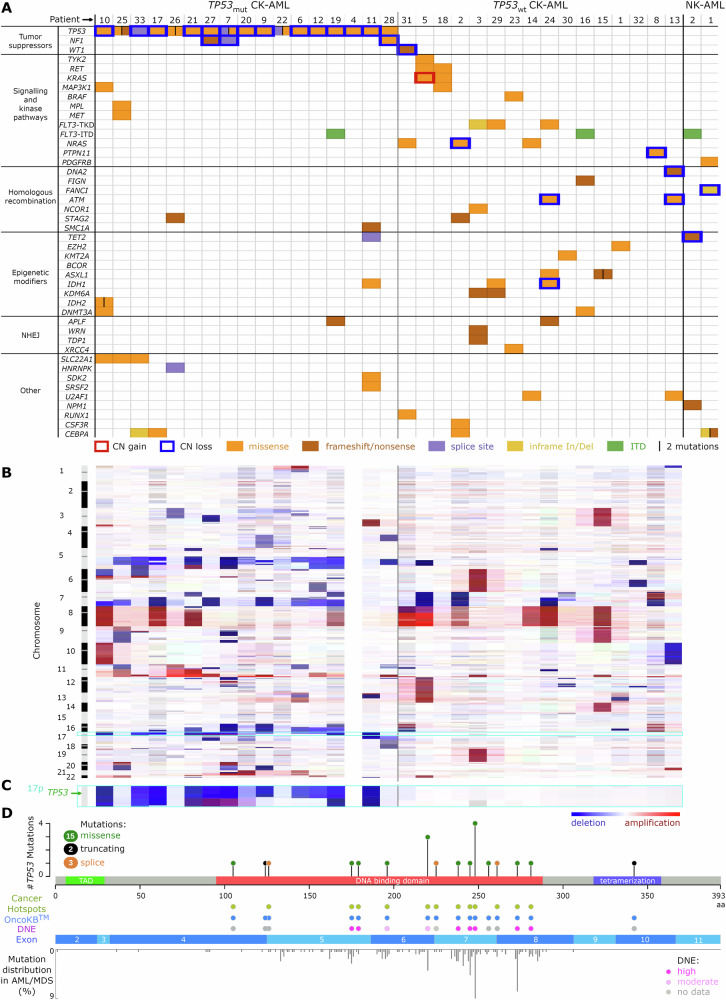
Dependence of the CK-AML mutational and copy number landscape on the *TP53* status. **A** Mutations in known cancer genes. **B** CNV profiles. **C** Magnified view of the chromosome 17 p arm, harboring the *TP53* locus. **D** Mutations in *TP53*. *TP53*_mut_ / *TP53*_wt_ *TP53* mutated/wild-type, CK-AML / NK-AML normal/complex karyotype acute myeloid leukemia, MDS myelodysplastic neoplasia, NHEJ non-homologous end joining, CN copy number, In/Del insertion/deletion, ITD internal tandem duplication, DNE dominant negative effect, TAD transactivation domain, aa amino acid.

The CD34+ and CD34- cellular fractions obtained after FACS sorting had nearly identical mutations, variant allele frequencies (VAFs), and cytogenetic profiles, except for patient 7 (Fig. S5). The patients were screened for mutations in common AML and pan-cancer genes of interest (Table S4). On average, 2.4 genes per patient (bootstrap error = 0.27) were affected by mutations predicted to be severely or moderately pathogenic. Apart from the *TP53* gene and other tumor suppressors, most perturbations affected one allele only, with no evidence for a mutation or loss of the other allele (Fig. 1A). The *TP53*_mut_ CK-AML group had relatively few mutations outside the tumor suppressor gene category. The *TP53*_wt_ CK-AML group had only one tumor suppressor gene mutation. This cohort had signaling pathways, epigenetic modifiers, and DNA damage repair component genes affected instead (Fig. S6).

The most frequent copy number aberrations in the *TP53*_mut_ subgroup were -5q (13/17), -7q (8/17), +11q (10/17), -16q (7/17) and -17p (12/17) (Fig. 1B, S7). With reference to the combined next generation sequencing CNV and fluorescence in situ hybridization (FISH) karyotyping results, all patients in this subgroup exhibited typical karyotypes, characterized by the loss of genomic material from the 5q, 7q, and 17p regions [12]. The most frequent copy number abnormalities in the *TP53*_wt_ group were +8 (6/16), -7q (5/16), as well as copy number neutral translocations involving 11q23 (7/16) corresponding to the *KMT2A* locus. The latter were exclusively observed in the *TP53*_wt_ subgroup. More than half of the *TP53*_wt_ patients (9/16) had atypical karyotypes without -5q, -7q, or -17p lesions (Fig. 1B, S7).

Within the *TP53* positive subgroup, 16 of 17 patients had the *TP53* gene biallelically inactivated, either by a second-hit mutation (*n* = 3) or by a deletion of the 17p chromosomal region harboring the *TP53* locus (*n* = 13) (Fig. 1A, C). *TP53* mutations clustered in the DNA-binding domain, consistent with prior reports [7]. They were mostly recurrent, previously described, and classified as dominant negative (Fig. 1D, Table S5).

**Fig. 2:**
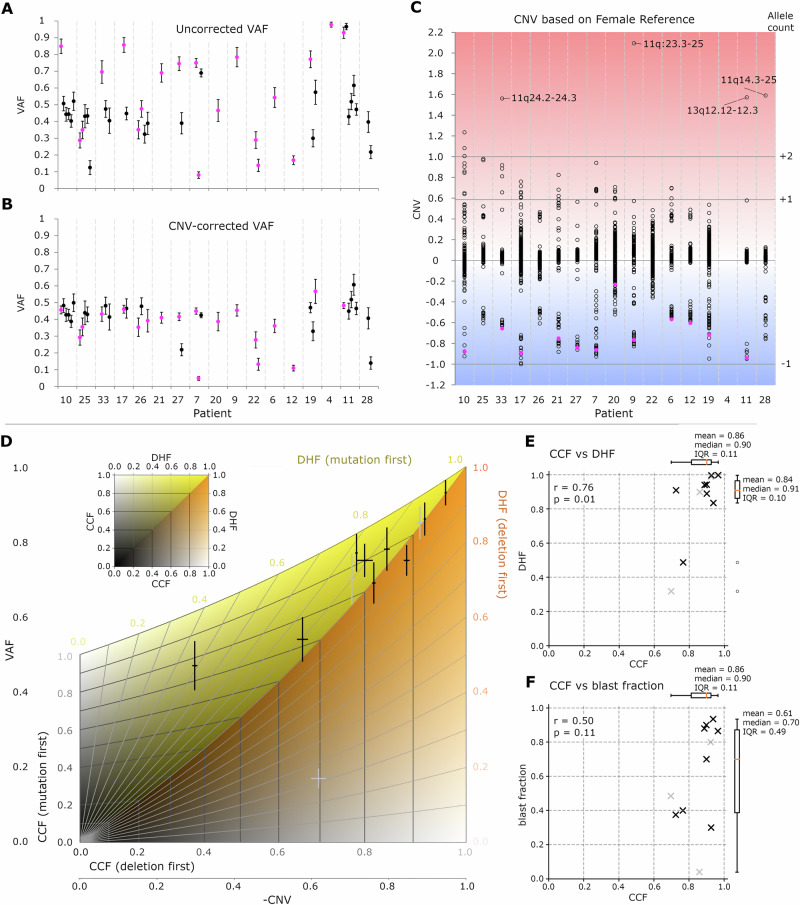
VAF and CNV analysis for the *TP53* mutated CK-AML patients. **A**, **B** Variant allele frequencies (*TP53* VAFs in pink): (**A**) raw, uncorrected VAFs. (**B**) VAFs corrected for the locus-specific copy number. **C** CNVs (*log*_*2*_*(allele count)-1*) for genomic segments (*TP53* containing segments in pink). Horizontal lines indicate expected CNV values for the loss of 1 allele, and gain of 1 or 2 alleles, for 100% penetrance. **D** Analysis of the *TP53* genotype for patients containing tumor clones with *TP53* mutation and deletion. **E**, **F** The CCF and DHF values were estimated from the -CNV and VAF values. **E** CCF versus DHF. **F** CCF versus blast fraction. In (**D**–**F**), data for untreated patients are in black, and those for treated patients are in grey. The vertical error bars correspond to the estimated standard deviations in (**A**, **D**) and the horizontal error bars reflect the differences in CNVs versus male and female references in D. The formulae for the error bars in (**B**) are listed in Suppl. Methods. VAF variant allele frequency, CNV copy number variation, CCF cancer clone fraction, DHF double hit fraction, IQR interquartile range.

Variant allele frequencies (VAF) were used to assess mutational burden (Fig. S8). *TP53* mutation VAFs were greater than VAFs of the other mutations found in the same patient (*p* = 8 × 10^−^^3^) (Fig. 2A). In the absence of karyotype changes, a higher VAF indicates a larger fraction of cells affected by a mutation, and thus an earlier mutational event. In the case of *TP53* mutations, however, VAFs were in most cases elevated in consequence of concurrent deletion of the other allele, which reduced the wild-type allele count. We computationally corrected this effect by calculating a VAF_corrected_ = VAF_observed_ × 2^CNV^ that would be observed if the same count of mutated alleles were present, but the deletion had not occurred. With this correction, the VAF values for the *TP53* mutations were no longer higher compared to other mutations (*p* = 0.56), arguing against a model of early occurrence of the *TP53* defects (Fig. 2B).

We then examined the genome-wide copy number variation (CNV) profiles (Fig. 1B). CNV values for *TP53* deletions were among the most negative CNVs in our cohort (*p* = 1 × 10^−^^4^ and *p* = 1 × 10^−^^3^ for female and male reference, respectively) (Fig. 2C, S9). Assuming that each deletion reflects the loss of one chromosomal copy, the large negative CNV values suggest a higher proportion of cells harboring *TP53* deletions. The CNV values for gains were typically too high to be explained by a single-allele gain, even if all cells carried the alteration, suggesting gains of multiple alleles (Fig. 2C).

From combined CNV and VAF data, it is possible to determine whether *TP53* mutations preceded or followed *TP53* locus deletions. Qualitatively, a high VAF speaks for the ‘mutation first’ scenario, whereas a high negative CNV advocates for a ‘deletion first’ scenario. However, because deletions also increase the VAF value, a mathematical model was necessary to draw definitive conclusions. We defined the cancer cell fraction (CCF) as the proportion of cells lacking a wt/wt *TP53* and the double hit fraction (DHF) as the subset of CCF cells with biallelic *TP53* inactivation. Negative CNV (-CNV) and VAF values were derived from CCF and DHF using analytical formulas for the ‘mutation-first’ and ‘deletion-first’ scenarios (see Supplementary Methods and Scheme S1). By varying CCF and DHF between 0 and 1, we determined the (non-overlapping) areas in the -CNV *versus* VAF plot that correspond to the mutation-first (yellow in Fig. 2D) and deletion-first (orange in Fig. 2D) scenarios. The dividing line between the two regions corresponds to the scenario where the sample contains only wt/wt cells and cells with a mutation and deletion, i.e., the scenario where mutation and deletion always co-occur. The -CNV *vs* VAF values for our patients mapped to the mutation-first region, rather than the deletion-first region, with uncertainty that could be explained by experimental errors (Fig. 2D, S10, 11, Table S6). Their proximity to the boundary suggests that the loss of the second allele rapidly followed *TP53* mutations. The only clear outlier to the rule was a pretreated patient for whom the *TP53* deletion clearly preceded the mutation of the other allele.

While CNV and VAF could be analytically calculated from CCF and DHF, analytic expressions for the reverse calculation could not be derived. However, it was possible to numerically invert the non-linear system of equations and deduce CCF and DHF from the experimental CNV and VAF data (Fig. 2E, Table S7). The results confirmed that DHF tended to be close to 1 for most patients, supporting the scenario of a rapid succession of *TP53* mutations and deletions. We also tested the possible dependence of the blast fraction on CCF, but detected no clear correlation (Fig. 2F).

In summary, our findings provide insight into the mechanism of karyotype decline in *TP53*-deficient CK-AML. We conclude that in CK-AML patients, *TP53* mutations can arise in any sequence relative to other driver mutations, but are rapidly followed by loss of the second *TP53* allele and subsequent karyotype decline. Our data also explain why most *TP53* mutations in CK-AML are dominant negative. Only such mutations drive loss of the remaining allele, facilitate subsequent karyotype aberrations, and promote disease progression. Our ‘mutation-first, then deletion’ model aligns with observations in acute leukemias and myelodysplastic syndromes [13], whereas the reverse sequence has been suggested for chronic lymphocytic leukemia and myeloma [14, 15].

## Supplementary information

Supplementary Methods, Tables, and Figures

Supplementary code for Fig. 2D-F

## Data Availability

The data generated and analyzed in the current study are available in the https://www.ncbi.nlm.nih.gov repository under BioProject ID PRJNA1256222.
