# Supplementary material for: Etiology of TP53 mutated complex karyotype acute myeloid leukemia
Source: Leukemia. 2025 Dec 19;40(2):444–8. doi: 10.1038/s41375-025-02835-9 (PMC12875860; doi:10.1038/s41375-025-02835-9)
Supplement: Supplementary file 1 — Supplementary Methods, Tables, and Figures [file 41375_2025_2835_MOESM1_ESM.pdf]

Supplementary Material
for

Etiology of *TP53* mutated complex karyotype acute myeloid leukemia

Anna Fedenko[1], Honorata Czapinska[1], Alwin Krämer[2,3],

Friedrich Stölzel[4,5], Tilmann Bochtler[2,3,6], Matthias Bochtler[1,7 §]

[1]*International Institute of Molecular and Cell Biology, Warsaw, Poland*

[2]*Clinical Cooperation Unit Molecular Hematology/Oncology, German Cancer Research Center (DKFZ), Heidelberg, Germany*

[3]*Department of Internal Medicine V, University of Heidelberg, Heidelberg, Germany*

[4]*Department of Hematology and Oncology, Universitätsklinikum Carl Gustav Carus an der Technischen Universität Dresden, Dresden, Germany*

[5]*Division of Stem Cell Transplantation and Cellular Immunotherapy, University Hospital Schleswig-Holstein, Campus Kiel, Kiel, Germany*

[6]*Department of Medical Oncology, National Center for Tumor Diseases, Heidelberg University Hospital, Heidelberg, Germany*

[7]*Institute of Biochemistry and Biophysics PAS, Warsaw, Poland*

[§]Corresponding author

Tel: +48225970732

e-mail: mbochtler@iimcb.gov.pl

Supplementary Methods

I. Analytical methods

***CCF* and *DHF* calculations:** We define the Cancer Cell Fraction (CCF) as the proportion of cells that are not wild-type at both *TP53* alleles,[1] and the Double Hit Fraction (DHF) as the proportion of CCF cells with alterations in both alleles (Scheme S1). In cells harboring a mutation and deletion, either event may occur first.

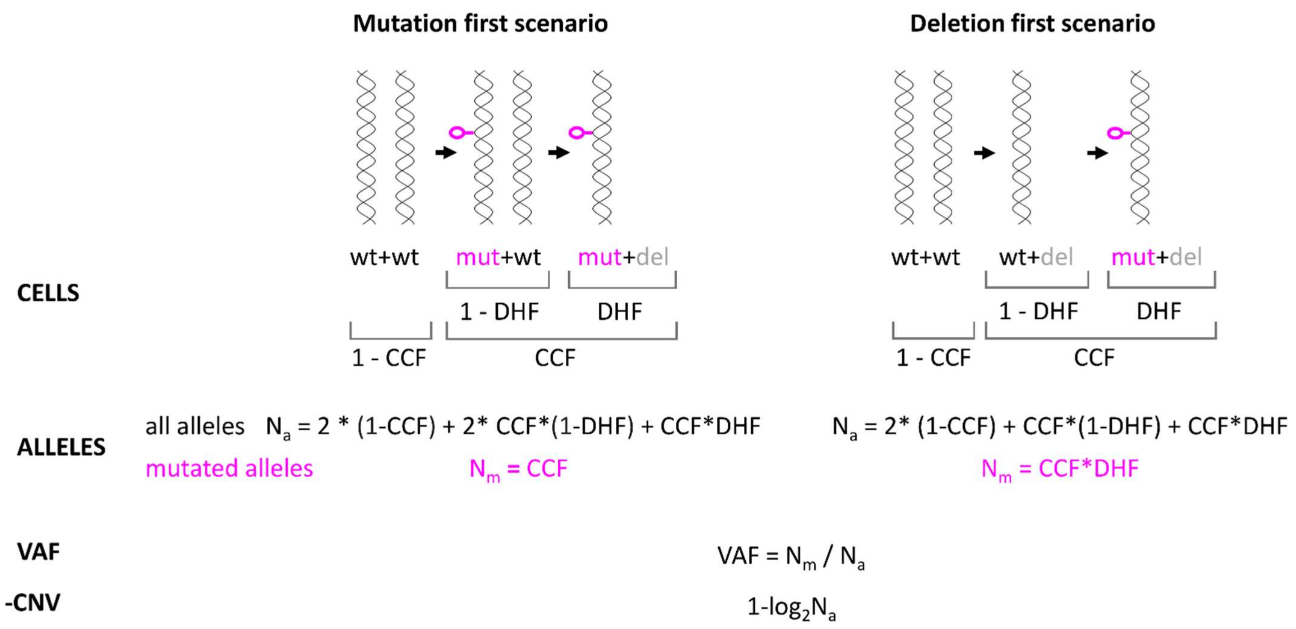

Scheme S1. Schematic illustration of Cancer Cell Fraction (CCF) and Double Hit Fraction (DHF) calculations based on number of all/mutated alleles (N_a, N_m), variant allele frequency (VAF) and copy number variation (CNV).

Qualitatively, if the mutation occurs early and the deletion late, the variant allele frequency (VAF) tends to be high, whereas the copy number variation (CNV) close to 0. By contrast, if the deletion occurs early and the mutation late, the $-CNV$ tends to be large and VAF close to 0. By varying CCF and DHF between 0 and 1, we determined the areas in the $-CNV/VAF$ plot that are compatible with the "mutation first" and "deletion first" scenarios (yellow and orange in Fig. 2D and S10A). The lines radiating from the origin indicate the DHF, in steps of 0.1. The mirrored L-shape lines indicate the CCF in steps of 0.1. The line between the yellow and orange areas relates VAF and CNV values for cases with only wt/wt or double-hit (mutation and deletion) cells in the sample (DHF of 1).

An analytic solution to express CCF and DHF in terms of $-CNV$ and VAF was not found. However, numerically the equations could be inverted to calculate CCF and DHF from the experimentally accessible $-CNV$ and VAF (Fig. 2EF and S10).

***CNV* corrected *VAF* values:** For a fixed fraction of mutated *TP53* in a cell population, the additional presence of deletions increases the measured VAF because it reduces the number of wild-type alleles. The VAF frequency that would be observed if all deleted alleles were wild-type equals:

$$VAF_{corr} = VAF_{meas} \times 2^{CNV}$$

The CNV values are negative for deletions, and therefore the 2^{CNV} values are positive and smaller than 1, and thus the corrected VAF values (VAF_{corr}) are lower (Fig. 2B).

Error estimation: To estimate the errors of VAF_{meas} (Fig. 2A, and vertical in Fig. 2D and S10), we used the Bernoulli expression for the standard deviation, $\sigma = \sqrt{np(1-p)}$, of the number of times a given allele (from two alternatives) is picked when the probability of picking the allele is p, and n choices are made. We expect the allele to be picked np times, and therefore the relative error for the number of picks to be: $\frac{\Delta(np)}{np} = \frac{\sigma}{np} = \sqrt{\frac{1-p}{np}}$. By identifying n with the total number of alleles N_a and approximating p by VAF_{meas}, we obtain:

$$\frac{\Delta VAF_{meas}}{VAF_{meas}} = \sqrt{\frac{1 - VAF_{meas}}{N_a \times VAF_{meas}}}$$

The error bars for the CNV values (horizontal in Fig. 2D and S10) were calculated as a difference between the CNV values obtained with male and female references:

$$\Delta CNV = |CNV_{FR} - CNV_{MR}|$$

where CNV_{FR} and CNV_{MR} denote the copy number variation estimated versus female and male references, respectively.

To obtain the error of VAF_{corr} (Fig. 2B), we used the rule that for a product, the relative errors add in quadrature:

$$\frac{\Delta VAF_{corr}}{VAF_{corr}} = \sqrt{\left(\frac{\Delta VAF_{meas}}{VAF_{meas}}\right)^2 + \left(\frac{\Delta\left(2^{CNV}\right)}{2^{CNV}}\right)^2}$$

To evaluate the second term under the root, we used the expression for the derivative of the natural logarithm, $\frac{dx}{x} = d(\ln x)$:

$$\frac{\Delta(2^{CNV})}{2^{CNV}} = \Delta\left(\ln\left(2^{CNV}\right)\right) = \Delta(CNV \times \ln 2) = \Delta CNV \times \ln 2$$

***VAF* and *CNV* values weighted based on CD34+ and CD34- fractions (for sorted samples):** Assuming that the fractions of CD34+ and CD34- cells in a sample determined by flow cytometry are c_{CD34+} and c_{CD34-}, the net (weighted) values for VAF and CNV are:

$$VAF_{net} = \frac{VAF_{CD34-} \times c_{CD34-} + VAF_{CD34+} \times c_{CD34}}{c_{CD34} + c_{CD34}}$$

$$CNV_{net} = log_2 \frac{c_{CD34-} \times 2^{CNV_{CD34-}} + c_{CD34+} \times 2^{CNV_{CD34+}}}{c_{CD34-} + c_{CD34+}}$$

The ΔVAF_{CD34-} and ΔVAF_{CD34+} errors should be uncorrelated, therefore ΔVAF_{net} should be:

$$\Delta VAF_{net} = \sqrt{(\Delta VAF_{CD34-} \times \frac{c_{CD34}}{c_{CD34-} + c_{CD34}})^2 + (\Delta VAF_{CD34+} \times \frac{c_{CD34+}}{c_{CD34} + c_{CD34}})^2}$$

For the CD34 sorted samples, the CNV values of CD34+ fractions are shown in Fig. 2C, and the weighted averages of VAFs and CNVs are shown in Fig. 2ABD and S10A. For patient 20, CNV and B-allele-based chromosome segmentation were inconsistent (Fig. S11).

II. Experimental methods

Patients and samples: AML patient samples were obtained from the Medical Faculty of the University of Heidelberg, the German Cancer Research Center (DKFZ), Heidelberg (9 CK-AML, 2 NK-AML patients), and the Medical Faculty Carl Gustav Carus, Technical University in Dresden, Germany (24 CK-AML patients). Patients gave informed consent to collect and share clinical material following ethical board approval in accordance with the Declaration of Helsinki (Ethics permits EK 98032010 from the Faculty of Medicine Carl Gustav Carus, Technical University in Dresden, and S-206/2011 from the Medical Faculty of the University of Heidelberg). Patient data and the corresponding samples were anonymized. The sequencing data submitted to the NCBI SRA database (ID: PRJNA1256222) were further anonymized with orthogonal IDs. The samples originated from mononuclear cell fractions of whole blood or bone marrow and were obtained as frozen cellular pellets, cryopreserved cells, or isolated genomic DNA (Table S1). They were kept at -80 °C for long-term storage and thawed directly before use.

Cytogenetics: The multicolor fluorescent in situ hybridization (FISH) and karyotype analysis were performed by the Institute of Human Genetics, University of Heidelberg, and the Dresden Hematology Unit, as described previously.[2] The scores for chromosomal abnormalities per patient were obtained as outlined by Grimwade et al. (2010).[3]

CD34 marker-based sorting: Fluorescence-Activated Cell Sorting (FACS) was performed for samples from patients 3, 5, 7, 10, and 19 (Table S1). Frozen samples were thawed in a water bath at 36 °C, and washed in 5% bovine serum albumin (BSA) in phosphate-buffered saline (PBS) by centrifuging 2 times for 5 minutes at 4 °C and 800 *g*. The cells were resuspended in 0.1 ml of PE(phycoerythrin)-conjugated anti-human CD34 antibody or PE-conjugated isotype control antibody diluted in 5% BSA in PBS (reagent and equipment details are listed in Table S8). Approximately 10^6 cells per antibody staining reaction were used. The samples were incubated in the dark, at room temperature for 30 min, washed twice, and resuspended in 0.5 ml of 5% BSA in PBS, 2 mM EDTA (ethylenediaminetetraacetic acid). Cell sorting was carried out using a FACS Aria II cell sorter with an 85 µm nozzle. Fluorescence was excited with a 488 nm laser (13 mW) and detected with a PE (585/42) emission filter. The samples stained with the isotype control antibody were used to determine the fluorescence background. Sorted fractions were immediately spun down for genomic DNA (gDNA) extraction. For samples sorted according to CD34 status, the results for the CD34+ fraction are shown (except for patient 19, for whom the CD34- fraction analysis was presented because analysis of the CD34+ fraction failed).

gDNA isolation: gDNA was extracted using the DNeasy Blood & Tissue Kit or Monarch Genomic DNA Purification Kit. gDNA was verified on a 1% agarose gel, and quality and concentration were assessed using Nanodrop and Quantus Fluorometer with QuantiFluor double stranded DNA dye.

Whole Exome Sequencing: Whole-exome next-generation sequencing (NGS) libraries were prepared using SureSelectXT HSQ Reagent Kit. For the CD34-sorted samples, 20 ng of gDNA was used as input due to limited gDNA availability. CD34+ and CD34- fractions were processed separately in all subsequent steps. 0.2 µg of gDNA was used for the CD34-unsorted patient samples. Target enrichment of libraries was done using OneSeq 300kb BB+Human All Exon V7 kit with baits targeting exons, high minor allele frequency SNP regions, and ClinGen defined[4] disease-associated regions (Fig. S1). The libraries were PCR-amplified. NGS was performed on the Illumina NextSeq (for patients 7 and 17) and the Illumina NovaSeq 6000 platform, with 2 x 100 cycles and paired-end mode. The libraries were sequenced with 50-100 million reads per sample, aiming for an average depth of 100x in the covered regions.

Genome alignment, variant calling, and annotation: The raw paired-end reads were quality-filtered, adaptor-trimmed, and aligned to the human genome assembly GRCh37 (hg19) using the BWA-MEM algorithm.[5] PCR duplicates were removed within the Agilent SureCall data analysis software (Table S9). The sorting and indexing steps were done using SAMtools[6] and variant calling using BCFtools[6] mpileup function to generate genotype likelihoods, which were then streamed into the BCFtools variant caller function. The raw .vcf files were quality filtered, whereby the variant calls with a quality value < 30 and the number of variant allele-supporting reads < 6 were removed. The remaining variants were submitted to the Variant Effect Predictor (VEP) server[7] for annotation.

Identification of driver mutations: The annotated variants affecting particular AML and pan-cancer genes of interest (Table S4) were obtained from VEP output. Only variants affecting exon and splice site regions ('moderate' and 'high' significance according to VEP) were considered, and only variants with allele frequencies worldwide (AF) < 5%

according to dbSNP,[8] as well as previously unreported variants, were used. The variants were queried for ClinVar[9] annotation. All 'Benign' and 'Likely benign' variants, as reported in ClinVar, were not considered further, and all 'Pathogenic' and 'Likely pathogenic' variants were deemed to be driver mutations. Additionally, a custom workflow outlined below was used to predict the probability of previously uncharacterized variants being pathogenic (Fig. S2).

Assessment of the pathogenicity of new and uncharacterized variants: To assess the likelihood of an SNP being pathogenic, the two bioinformatic tools, AlphaMissense[10] and ESMVariants,[11] were used in combination. If both tools marked an SNP as pathogenic or benign, the variant was considered pathogenic or benign, respectively. If the two tools produced discrepant interpretations, the AlphaMissense outcome was applied. The variant was considered pathogenic if it was purely somatic as defined by VEP and if at least one of the above tools called it pathogenic. All frameshift and inframe indel variants were deemed pathogenic in the context of this classification (Fig. S2). All mutations reported in this study were validated using the Integrative Genomics Viewer (IGV)[12].

FLT3-ITD detection: Detection of the internal tandem duplications in the *FLT3* gene was performed using the FLT3-ITDetect software.[13]

CNV analysis: The copy number variation profile for each patient was analyzed using EXCAVATOR2,[14] which performs genome segmentation and calls CNVs on whole exome sequencing data using both in- and off-target reads. The analysis was carried out in the 'pooling' mode, and Female and Male OneSeq DNA samples (Table S8) were used as references. The regions covered by the OneSeq 300kb BB+Human All Exon V7 probes were specified as the 'in-target' ones, the window size was set to 10 kB, and the hg19 assembly was applied. The Female OneSeq Human Reference DNA was used as a control for the CNV analysis presented in Fig. 1BC and 2C, the Male OneSeq Human Reference DNA in Fig. S9. The analysis for patient 4 failed and is thus not shown.

The output from EXCAVATOR2 was converted to the GLAD format, and minor overlaps between segments were removed by manual adjustment of segment coordinates. Consistency between the results obtained using the two references was checked as outlined in Fig. S3. Segments shorter than 3 Mb were classified as noise and filtered out. The formatted and filtered segmentation files for each patient were concatenated and supplied to the GISTIC2.0 module[15] of the GenePattern server to perform the meta-analysis and visualization.

Supplementary Tables

Table S1

Patient ID	CD34 (%)	Blasts (%)	Source	Material	Sex
CK-AML patient 1	0	86	bone marrow	cryopreserved cells	m
CK-AML patient 2	41	44	bone marrow	DNA	f
CK-AML patient 3 [a]	76.2	94.5	bone marrow	cryopreserved cells	f
CK-AML patient 4	94	89	bone marrow	cryopreserved cells	m
CK-AML patient 5 [a]	38	17	bone marrow	cryopreserved cells	f
CK-AML patient 6	91	37.5	bone marrow	DNA	f
CK-AML patient 7 [a]	56	70	peripheral blood	cryopreserved cells	f
CK-AML patient 8	14	44	bone marrow	DNA	m
CK-AML patient 9	84	90	bone marrow	DNA	m
CK-AML patient 10 [a,t]	30	80	bone marrow	cryopreserved cells	f
CK-AML patient 11	75	86.5	bone marrow	cryopreserved cells	m
CK-AML patient 12 [t]	79	48.5	bone marrow	DNA	f
CK-AML patient 13	0.1	41	peripheral blood	DNA	m
CK-AML patient 14	0.4	84	bone marrow	cryopreserved cells	m
CK-AML patient 15	0	91	bone marrow	cryopreserved cells	m
CK-AML patient 16	21.5	34	bone marrow	DNA	f
CK-AML patient 17	71	30	bone marrow	DNA	m
CK-AML patient 18	15	58	bone marrow	cryopreserved cells	f
CK-AML patient 19 [a]	19	93.5	bone marrow	cryopreserved cells	m
CK-AML patient 20	100	40	bone marrow	DNA	m
CK-AML patient 21	75	nd	bone marrow	DNA	m
CK-AML patient 22	38	34	bone marrow	DNA	m
CK-AML patient 23	0	93	bone marrow	cryopreserved cells	f
CK-AML patient 24	3.4	95	bone marrow	cryopreserved cells	f
CK-AML patient 25	69	nd	bone marrow	DNA	m
CK-AML patient 26 [t]	30	41.79	bone marrow	DNA	f
CK-AML patient 27	87	88	bone marrow	cryopreserved cells	m
CK-AML patient 28	19	47	bone marrow	DNA	f
CK-AML patient 29	nd	80	bone marrow	cryopreserved cells	m
CK-AML patient 30	65	29.5	bone marrow	DNA	m
CK-AML patient 31	41	86	bone marrow	cryopreserved cells	f
CK-AML patient 32	99.6	90	bone marrow	cryopreserved cells	f
CK-AML patient 33 [t]	6	4	bone marrow	cryopreserved cells	m
NK-AML patient 1	92	98	bone marrow	frozen pelleted cells	m
NK-AML patient 2	100	0.2	peripheral blood	frozen pelleted cells	f

Table S1. CK-AML and control sample specification. [a] CD34 sorted AML samples; [t] AML secondary to prior treatment; nd - no data, m - male, f - female. CK/NK-AML – complex/normal karyotype acute myeloid leukemia.

Table S2

Patient ID	Age	Intensive Induction (yes = 1, no = 0)	CR yes= 1 no=0	*TP53* status (HTS)	Allogeneic transplant	Overall survival (months)	alive = 0 death = 1	progression-free survival (months)	PFS event (0=no, 1=yes)
**33**	58	0	0	mut	0	0.3?	1	not given	not given
**7**	77	0	0	mut	0	0.3	1	0.3	1
**10**	71	0	0	mut	0	NA	NA	NA	NA
**20**	72	0	0	mut	1	8.9	1	6.5	1
**11**	59	1	0	mut	0	0.1	1	0.1	1
**27**	63	1	0	mut	0	1.7	1	0.8	1
**19**	51	1	0	mut	0	2.4	1	0.2	1
**4**	72	1	0	mut	0	0.2	1	0.2	1
**9**	66	1	0	mut	0	2.3	1	1.6	1
**17**	53	1	1	mut	1	4.8	1	4.8	1
**12**	73	1	1	mut	0	12.0	1	9.6	1
**26**	49	1	0	mut	1	3.2	1	0.3	1
**6**	72	1	0	mut	0	7.7	1	2.6	1
**21**	58	1	1	mut	1	5.5	1	3.3	1
**22**	69	1	1	mut	0	15.9	1	6.2	1
**25**	73	1	1	mut	0	7.7	1	7.7	1
**28**	47	1	1	mut	1	10.7	1	10.1	1
**Total number**		**13**	**6 (46% CR after intensive induction)**						
**5**	85	0	0	wt	0	2.3	1	2.3	1
**18**	66	0	0	wt	0	3.3	1	0.8	1
**13**	73	0	0	wt	0	1.4	1	1.4	1
**31**	30	1	1	wt	1	104.5	0	45.5	1
**3**	43	1	1	wt	1	97.8	0	97.8	0
**1**	68	1	0	wt	0	6.8	1	1.2	1
**14**	40	1	1	wt	1	8.4	1	6.3	1
**23**	39	1	0	wt	0	0.1	1	0.1	1
**29**	32	1	1	wt	0	4.5	0	4.5	0
**32**	42	1	0	wt	1	8.5	0	1.3	1
**24**	22	1	0	wt	1	26.8	1	0.2	1
**15**	53	1	1	wt	1	25.7	0	14.7	1
**2**	55	1	0	wt	1	49.3	1	1.0	1
**30**	61	1	0	wt	0	17.7	1	0.9	1
**8**	26	1	1	wt	1	15.2	0	14.5	1
**16**	39	1	0	wt	1	22	1	11.0	1
**Total number**		**13**	**6 (46% CR after intensive induction)**						

Table S2. Treatment details for the CK-AML patients in the *TP53*$_{mut}$ and *TP53*$_{wt}$ groups. CR – complete remission, PFS – progression-free survival, HTS – high-throughput sequencing, mut – mutated, wt – wild type.

Table S3

Sample	Reads passing mapping quality filters [10^6]	Reads in covered regions [10^6]	Reads in covered regions [%]	Target regions covered by at least 100 reads [%]	Covered regions with zero coverage [%]	High quality mapped bases [10^9]	Bases in target regions [10^9]	Average read depth in target regions	Median read depth in target regions
OneSeq Female Reference	167.63	120.19	71.70	92.27	2.77	16.93	9.94	161	154
OneSeq Male Reference	107.20	74.64	69.63	86.08	2.75	10.83	6.34	102	95
CK-AML patient 1	136.08	95.86	70.44	90.57	2.45	13.73	7.93	128	120
CK-AML patient 2	96.08	64.94	67.59	80.39	3.1	9.69	5.40	87	80
CK-AML patient 3 CD34+	121.53	78.28	64.41	90.39	2.96	12.27	6.43	104	102
CK-AML patient 3 CD34-	124.95	76.44	61.18	90.28	2.85	12.61	6.21	100	100
CK-AML patient 4	279.17	183.48	65.72	82.21	15.52	28.16	14.82	240	251
CK-AML patient 5 CD34+	140.55	98.95	70.40	90.18	2.97	14.17	8.23	133	128
CK-AML patient 5 CD34-	59.87	36.29	60.62	50.41	3.28	6.04	2.96	47	47
CK-AML patient 6	101.20	74.71	73.82	84.62	3.07	10.21	6.19	100	91
CK-AML patient 7 CD34+	112.09	70.16	62.60	87.34	2.9	11.31	5.71	92	91
CK-AML patient 7 CD34-	109.69	69.28	63.16	85.09	3.01	11.07	5.64	91	90
CK-AML patient 8	94.36	60.08	63.67	76.25	2.7	9.52	4.97	80	72
CK-AML patient 9	104.11	72.28	69.42	81.32	2.58	10.49	5.96	96	85
CK-AML patient 10 CD34+	95.39	57.43	60.21	80.46	3.02	9.63	4.68	75	73
CK-AML patient 10 CD34-	113.53	72.61	63.96	88.03	2.99	11.46	5.94	96	94
CK-AML patient 11	146.13	103.05	70.52	90.78	2.37	14.74	8.39	136	128
CK-AML patient 12	86.00	63.35	73.67	79.87	3.24	8.67	5.27	85	78
CK-AML patient 13	105.20	74.75	71.05	83.58	2.85	10.61	6.21	100	91
CK-AML patient 14	134.04	96.82	72.23	90.7	2.5	13.53	8.01	129	121
CK-AML patient 15	128.93	90.07	69.86	89.98	2.51	13.01	7.38	119	112
CK-AML patient 16	102.63	75.80	73.86	86.37	3.05	10.36	6.29	101	94
CK-AML patient 17	103.15	68.37	66.28	80.56	2.71	10.40	5.72	92	83
CK-AML patient 18	140.24	100.53	71.69	91.5	2.85	14.15	8.27	134	127
CK-AML patient 19 CD34-*	111.36	73.36	65.88	87.67	2.67	11.19	6.07	98	96
CK-AML patient 20	95.88	60.47	63.07	75.49	2.73	9.67	5.03	81	73
CK-AML patient 21	108.56	78.41	72.23	83.61	2.68	10.96	6.52	105	94
CK-AML patient 22	96.14	59.18	61.56	75.94	2.46	9.70	4.85	78	71
CK-AML patient 23	138.92	103.33	74.38	91.24	2.99	14.02	8.63	139	130
CK-AML patient 24	117.44	78.93	67.21	86.16	2.92	11.85	6.55	106	97
CK-AML patient 25	97.10	71.97	74.12	83.26	2.72	9.80	6.00	97	88
CK-AML patient 26	93.05	70.28	75.53	82.91	3.21	9.39	5.85	94	86
CK-AML patient 27	149.19	111.04	74.43	91.27	2.58	15.06	9.27	150	140
CK-AML patient 28	88.77	65.13	73.37	79.68	3.2	8.96	5.42	87	78
CK-AML patient 29	128.42	90.69	70.62	90.1	2.5	12.95	7.52	121	114
CK-AML patient 30	96.32	71.47	74.20	83.75	2.69	9.71	5.99	97	88
CK-AML patient 31	129.07	83.69	64.84	89.2	2.91	13.02	6.88	111	106
CK-AML patient 32	151.00	103.18	68.33	91.48	2.78	15.24	8.44	136	132
CK-AML patient 33	133.34	95.10	71.32	90.71	2.48	13.46	7.84	127	121
NK-AML patient 1	99.95	67.53	67.57	81.93	2.74	10.08	5.64	91	83
NK-AML patient 2	101.51	67.53	66.53	83.25	3.07	10.23	5.61	90	83

Table S3. Sequencing data quality. * The analysis with the SureCall software was unsuccessful for the CD34+ fraction of cells from patient 19. CK/NK-AML – complex/normal karyotype acute myeloid leukemia.

Table S4

AML	*ASXL1, CSF3R, CBL, CEBPA, DNMT3A, EZH2, FLT3, IDH1, IDH2, JAK2, MPL, NPM1, NRAS, RUNX1, SETBP1, SF3B1, SF3B2, TET2, TP53, U2AF1, UTY, WT1, PTPN11, NF1, KRAS, KDM6A, KIT, BCOR, KMT2A*
PAN cancer	*HNRNPK, ABL1, AKT1, ALK, APC, BRAF, ABCB1, ABCC2, ABL2, AKT2, AKT3, ATRX, CDA, CDKN2B, CDH7, CHIK2, CREBBP, CRLF2, CSF1R, BRCA1, BRCA2, CDKN2A, CTNNB1, ERBB2, DDR1, DDR2, DDX3X, DPYD, ERBB3, ERBB4, ERG, ESR2, EGFR, ESR1, EGFR2, HRAS, FBXW7, FGFR1, FGFR3, FGFR4, FLT1, FLT4, FSTL5, GNA11, GNAQ, GNAS, GSTP1, H3F3A, HNF1A, KZF1, IL2RA, MAP2K2, MET, IL2RB, IL2RG, INPP4B, JAK1, JAK3, KDR, LAMA2, LCK, LTK, MAP2K1, MAP2K4, MAP3K1, MAPK1, MED13, MYC, MYD88, NOTCH1, MTOR, PDGFRA, MLH1, MST1R, NELL2, PDGFRB, PHF6, PIK3R1, PSMB1, PSMB5, PSMD1, PSMD2, RAF1, RARA, RARB, RARG, PIK3CA, PTCH1, RB1, RET, ROS1, RPS6KB1, RXRA, RXRB, RXRG, SHH, SHOC2, SLC22A1, SLC22A2, SLC31A1, SLC34A2, SLC45A3, SLCO1B1, SMAD4, FSMARCA4, SMO, STK11, VHL, SMARCB1, SNCAIP, SOS1, SPRED1, SRC, SUFU, TAS2R38, TRRAP, TYK2, UGT1A1, YES1, ZMYM3, SDK2*
Homologous recombination (HR)	*BARD1, RAD51, ELP4, FIGN, RAD51B, RAD51C, RAD51D, DMC1, XRCC3, NME1, H2AFX, RAB3GAP2, FANCD2, FANCI, RAN, RASEF, SMG1, ATM, ATR, MND1, PSMC3IP, UPF1, DNA2, WDR91, ZDHHC13, ARD1A, BRDT, CDH1, MSH2, LIG4, ERCC2, NCOR1, EXO1, PALB2, PTEN, SMC1A, SMC3, STAG2, RAD21, FAM5C*
Non-homologous end joining (NHEJ)	*XRCC6, XRCC5, XRCC7, PRKDC, XRCC4, 53BP1, PNKP, XLF, PAXX, APLF, WRN, MRI, CYREN, PNKP, TDP1, TDP2, APTX, POLD1, POLD2, POLD3, POLD4, POLM, DNTT, DCLRE1C, SETMAR*

Table S4. The list of studied gene panels and genes that were scanned for the presence of mutations in this study.

Table S5

Patient ID	Mutation type	VAF	CNV score	Mutation site	Dominant negative effect
CK-AML patient 21	missense	0.69	-0.751	G105V	nd
CK-AML patient 25	frameshift	0.35	0.025	T123X	nd
CK-AML patient 12	missense	0.17	-0.627	R175H	Y
CK-AML patient 27	missense	0.75	-0.842	H179R	Y
CK-AML patient 7	missense	0.08	-0.739	R196P	M
CK-AML patient 25	missense	0.29	-0.01	Y220C	M
CK-AML patient 9	missense	0.78	-0.789	Y220C	M
CK-AML patient 6	missense	0.54	-0.689	Y220C	M
CK-AML patient 17	missense	0.86	-0.985	C238Y	Y
CK-AML patient 22	missense	0.3	-0.06	G245S	Y
CK-AML patient 26	missense	0.35	0.01	R248Q	Y
CK-AML patient 28	missense	0.4	0.014	R248Q	Y
CK-AML patient 19	missense	0.77	-0.714	R248W	Y
CK-AML patient 4	missense	0.98	nd	R248W	Y
CK-AML patient 10	missense	0.85	-0.87	T256K	nd
CK-AML patient 11	missense	0.93	-0.946	R273C	Y
CK-AML patient 26	missense	0.47	0.01	D281Y	Y
CK-AML patient 20	stop gained	0.47	-0.266	R342*	nd
CK-AML patient 33	splice site	0.7	-0.688	splice acceptor (chr17:7578556-7578556)	
CK-AML patient 7	splice site	0.75	-0.739	splice acceptor (chr17:7577142-7577163)	
CK-AML patient 22	splice site	0.14	-0.06	splice acceptor (chr17:7577610-7577610)	

Table S5. Details on the *TP53* mutations reported in this study. The CNV score is the average of scores based on Male and Female References. For splice-site mutations, the chromosomal locations were provided relative to the hg19 human genome assembly. Positions, type, and significance of the detected *TP53* mutations were plotted with cBioPortal.[16] The dominant negative effect (DNE) of mutations was based on the IARC *TP53* database.[17] VAF – variant allele frequency, CNV – copy number variation, Y – full, M – moderate, nd - no data.

Table S6

Patient ID	*TP53* reference allele count	*TP53* alternative allele count	*TP53* total allele count	VAF	VAF std upper boundary	VAF std lower boundary	CNV vs FR autosomes only	CNV vs MR autosomes only	CNV vs FR default	CNV vs MR default	pre-treated
CK-AML 10 [a]	1	31	32	0.85	0.893	0.807	-0.872	-0.888	-0.878	-0.913	Y
CK-AML 33	14	32	46	0.7	0.768	0.632	-0.694	-0.714	-0.656	-0.716	Y
CK-AML 17	9	54	63	0.86	0.904	0.816	-0.893	-0.893	-0.891	-0.896	N
CK-AML 21	22	49	71	0.69	0.745	0.635	-0.766	-0.756	-0.752	-0.754	N
CK-AML 27	29	85	114	0.75	0.791	0.709	-0.854	-0.838	-0.844	-0.844	N
CK-AML 7 [a]	2	22	24	0.75	0.794	0.706	-0.716	-0.758	-0.706	-0.769	N
CK-AML 9	11	40	51	0.78	0.838	0.722	-0.787	-0.803	-0.769	-0.809	N
CK-AML 6	32	38	70	0.54	0.600	0.480	-0.562	-0.591	-0.568	-0.608	N
CK-AML 19 [n]	16	54	70	0.77	0.820	0.720	-0.719	-0.713	-0.718	-0.713	N
CK-AML 12	172	35	207	0.17	0.196	0.144	-0.599	-0.637	-0.604	-0.655	Y
CK-AML 11	4	53	57	0.93	0.964	0.896	-0.949	-0.944	-0.943	-0.945	N
CK-AML 20	31	27	58	0.47	0.535	0.405	-0.291	-0.304	-0.233	-0.302	N

Table S6. Data for VAF -CNV relationship calculation (input for Fig. 2D). The CNV values from EXCAVATOR were calculated either for autosomes only or in a default setting including chromosome X and smoothed. Weighted averages were used for the CD34 sorted samples except for patient 19. VAF – variant allele frequency, CNV – copy number variation, FR – OneSeq Female Reference, MR – OneSeq Male Reference, std – standard deviation. [a] CD34 sorted samples, [n] CD34- fraction used.

Table S7

Patient ID	blast fraction	pretreated	DNE	Mean -CNV	VAF	CCF	DHF	deletion fraction
CK-AML patient 10 [a]	80	Y	ND	0.880	0.85	0.924	0.989	0.913
CK-AML patient 33	4	Y	ND	0.704	0.7	0.859	0.898	0.772
CK-AML patient 17	30	N	Y	0.893	0.86	0.926	0.996	0.923
CK-AML patient 21	nd	N	ND	0.761	0.69	0.820	0.993	0.820
CK-AML patient 27	88	N	Y	0.846	0.75	0.887	0.940	0.887
CK-AML patient 7 [a]	70	N	ND	0.737	0.75	0.900	0.889	0.800
CK-AML patient 9	90	N	M	0.795	0.78	0.899	0.942	0.847
CK-AML patient 6	37.5	N	M	0.576	0.54	0.724	0.909	0.659
CK-AML patient 19 [n]	93.5	N	Y	0.716	0.77	0.938	0.835	0.782
CK-AML patient 12	48.5	Y	Y	0.618	0.17	0.697	0.318	0.697
CK-AML patient 11	86.5	N	Y	0.947	0.93	0.965	0.998	0.962
CK-AML patient 20	40	N	ND	0.297	0.47	0.765	0.487	0.373

Table S7. Data for CCF and DHF calculation (input for Fig. 2EF). The calculations were performed as outlined in Supplementary Methods. Mean -CNV was calculated as the average of values obtained for male and female references, with EXCAVATOR run for autosomes only. CCF – cancer clone fraction, DHF – double hit fraction. DNE – dominant negative effect (Y – full, M – moderate, ND – not determined), [a] CD34 sorted samples, [n] CD34- fraction used, nd – no data.

Table S8

Purpose	Reagent/Equipment	Supplier	Town, country	Cat. Number
**gDNA isolation**	DNeasy Blood & Tissue Kit	QIAGEN	Hilden, Germany	69504
	Monarch Genomic DNA Purification Kit	New England Biolabs	Ipswich, MA, USA	T3050
	QuantiFluor dsDNA Dye	Promega Corporation	Madison, WI, USA	E2670
**FACS**	PE anti-human CD34 Antibody (mouse IgG1, clone # 581) Dilution: 1 in 50	BioLegend	San Diego, CA, USA	343505
	Mouse IgG1 PE-conjugated Antibody (clone # 11711) Dilution: 1 in 100	Biotechne	Minneapolis, MN, USA	IC002P
	FACS Aria II cell sorter	Becton Dickinson	San Jose, CA, USA	
**Whole Exome Sequencing**	SureSelectXT HSQ Reagent kit	Agilent Technologies	Santa Clara, CA, USA	G9611A
	Herculase II Fusion DNA Polymerase kit	Agilent Technologies	Santa Clara, CA, USA	600677
	OneSeq 300kb BB+Human All Exon V7 kit	Agilent Technologies	Santa Clara, CA, USA	5191-4022
	Female OneSeq Human Reference DNA	Agilent Technologies	Santa Clara, CA, USA	51908848
	Male OneSeq Human Reference DNA	Agilent Technologies	Santa Clara, CA, USA	51908850
	NovaSeq 6000 S1 Reagent Kit	Illumina	San Diego, CA, USA	20028318
	Next Seq 500/550 High Output Kit (300 cycles)	Illumina	San Diego, CA, USA	20024908
	Quantus Fluorometer	Promega Corporation	Madison, WI, USA	TM369
	Ultrasonicator	Covaris	Woburn, MA, USA	E6150

Table S8. Reagents and equipment used in the study.

Table S9

Purpose	Software / database	Supplier	Town, country	Version
Sequencing data preprocessing	SureCall	Agilent Technologies	Santa Clara, CA, USA	4.2.1
	BWA MEM	Wellcome Sanger Institute (WSI)	Hinxton, Cambridge, UK	
	SAMtools	Genome Research Limited, WSI	Hinxton, Cambridge, UK	1.3
Variant calling and annotation	BCFtools	Genome Research Limited, WSI	Hinxton, Cambridge, UK	1.13
	Ensembl Variant Effect Predictor	EMBL, EBI	Hinxton, Cambridge, UK	
	dbSNP	NCBI, NIH	Bethesda, MD, USA	
	ClinVar	NCBI, NIH	Bethesda, MD, USA	
	AlphaMissense	DeepMind, Google	London, UK	
	ESMVariants	Ntranos Lab, UCSF	San Francisco, CA, USA	
	FLT3-ITDetect	Seoul National University	Seoul, South Korea	
Copy number analysis and visualization	EXCAVATOR2	University of Florence	Florence, Italy	1.1.2
	GISTIC2	GenePattern, MIT	Cambridge, MA, USA	2.0

Table S9. Software and databases used in the study.

Supplementary Figures

Figure S1

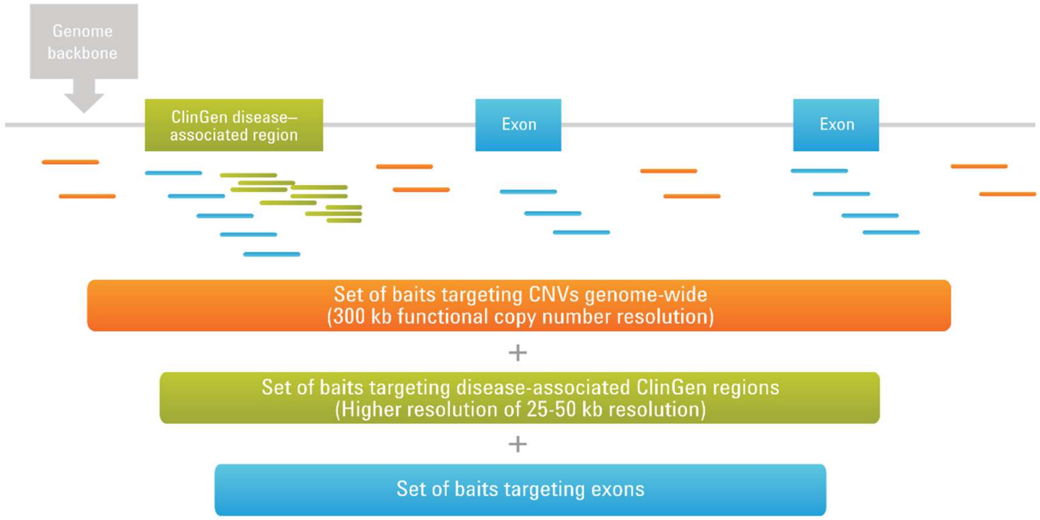

Figure S1. Schematic view of the OneSeq 300kb BB+Human All Exon V7 kit (Agilent Technologies). The enrichment scheme is based on three types of baits for: (i) genome-wide CNV and copy neutral loss of heterozygosity (cnLOH) detection (0.3 and 5 Mb resolution, respectively), (ii) ClinGen[4] disease-associated regions (25-50 kb resolution), and (iii) whole exome targeting with SureSelect Human All Exon V7 (adopted from the Agilent flyer). CNV – copy number variation, kb – kilobase.

Figure S2

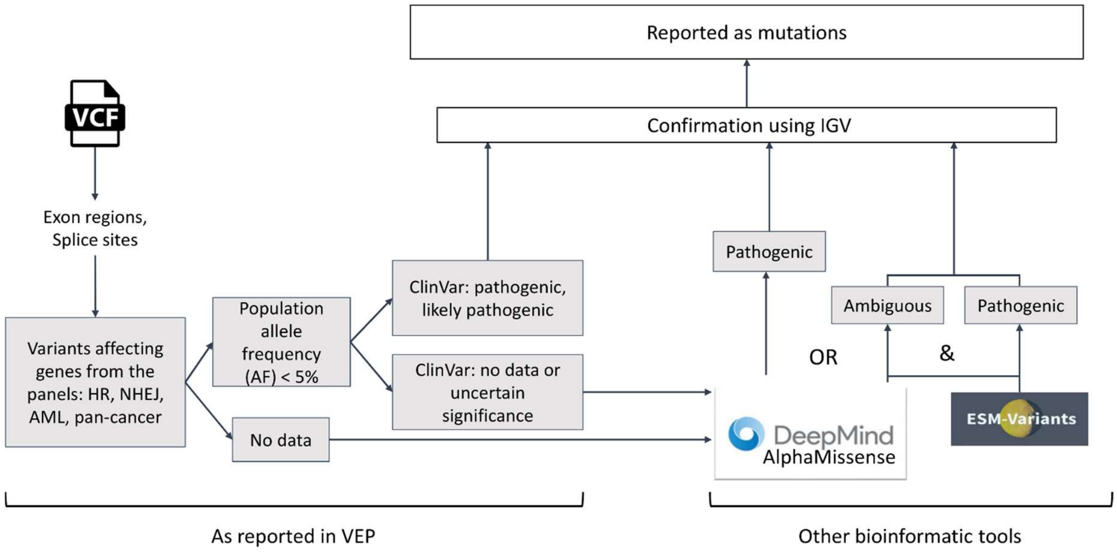

Figure S2. Flowchart of the significance analysis for observed mutations. Quality-filtered .vcf files were screened for variants in the preselected genes known to play a role in cancer, including but not limited to AML and genes from DNA damage repair panels (Table S4). Variants were retained for further analysis if they affected exons or splice sites, their allele frequency (AF) across the global population was < 5%, and they were designated as 'Pathogenic' and/or 'Likely pathogenic' in ClinVar.[9] The rare variants with no clear ClinVar designation were retained if they caused frameshift or in-frame insertions and deletions, were predicted 'pathogenic' by AlphaMissense,[10] were purely somatic according to VEP server,[7] and predicted 'pathogenic' by either AlphaMissense or ESM-Variants,[11] or were reported 'ambiguous' by AlphaMissense and 'pathogenic' by ESM-Variants. All reported variants were validated using IGV.[12]

Figure S3

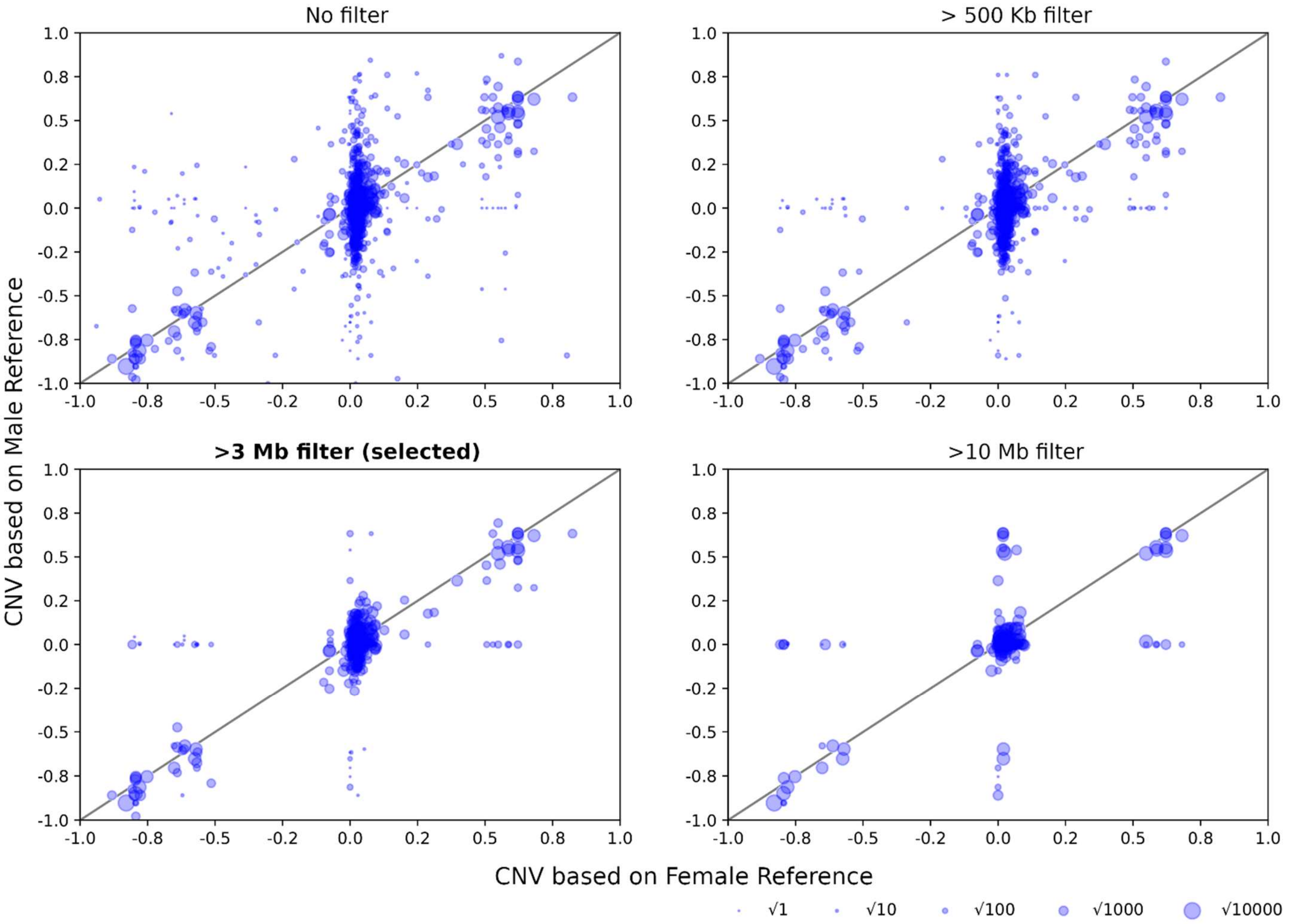

Figure S3. Comparison of copy number variation (CNV) data for a single patient (21), depending on the reference sample. CNV values based on male reference versus CNV values based on female reference, for regions greater than 0.1, 0.5, 3, and 10 Mb. For regions of 3 Mb or more, the CNV values were robust against the choice of reference (i.e. lie on the diagonal). Kb – kilobase, Mb – megabase.

Figure S4

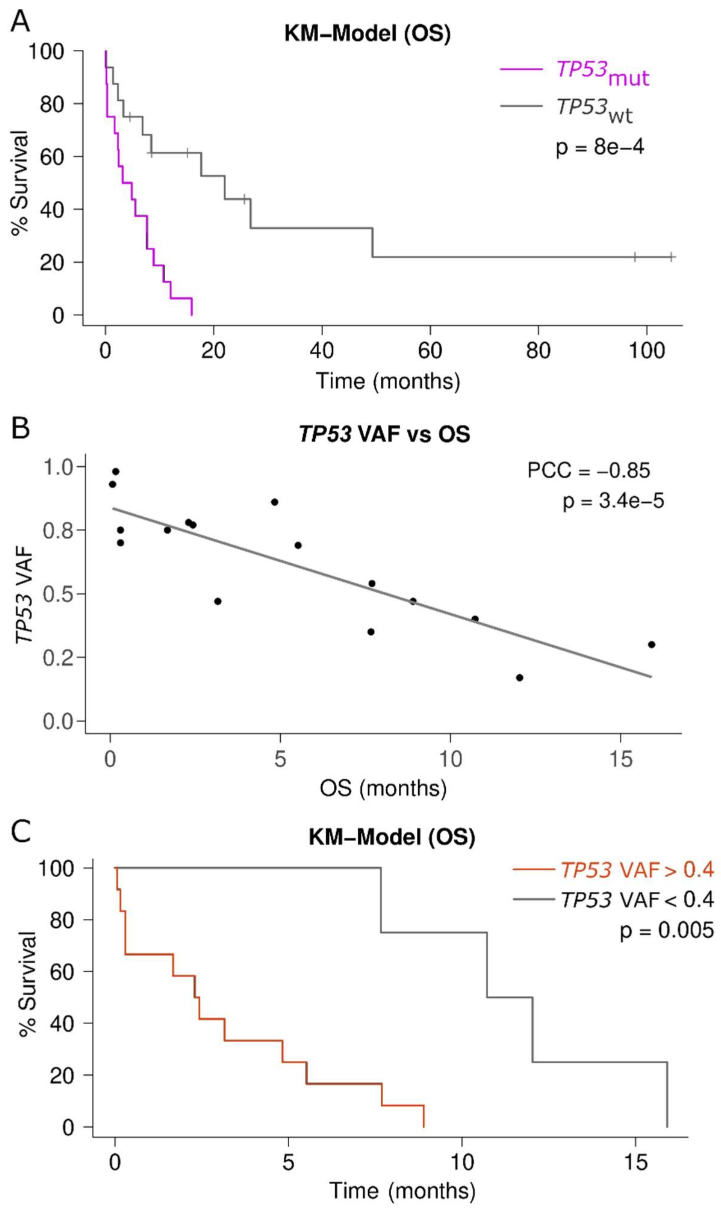

Figure S4. Variant allele frequencies (VAF) of CK-AML patients. (A) Kaplan-Meier (KM) survival analysis for *TP53* wild-type and mutated patients. **(B)** Correlation between *TP53* variant allele frequency (VAF) and the overall survival (OS). Upper VAF values were used for patients with more than one *TP53* mutation, and weighted averages of VAF values were used for the CD34-sorted samples. **(C)** Kaplan-Meier survival analysis for patients with *TP53* VAF above and below 0.4. PCC – Pearson correlation coefficient.

Figure S5

A

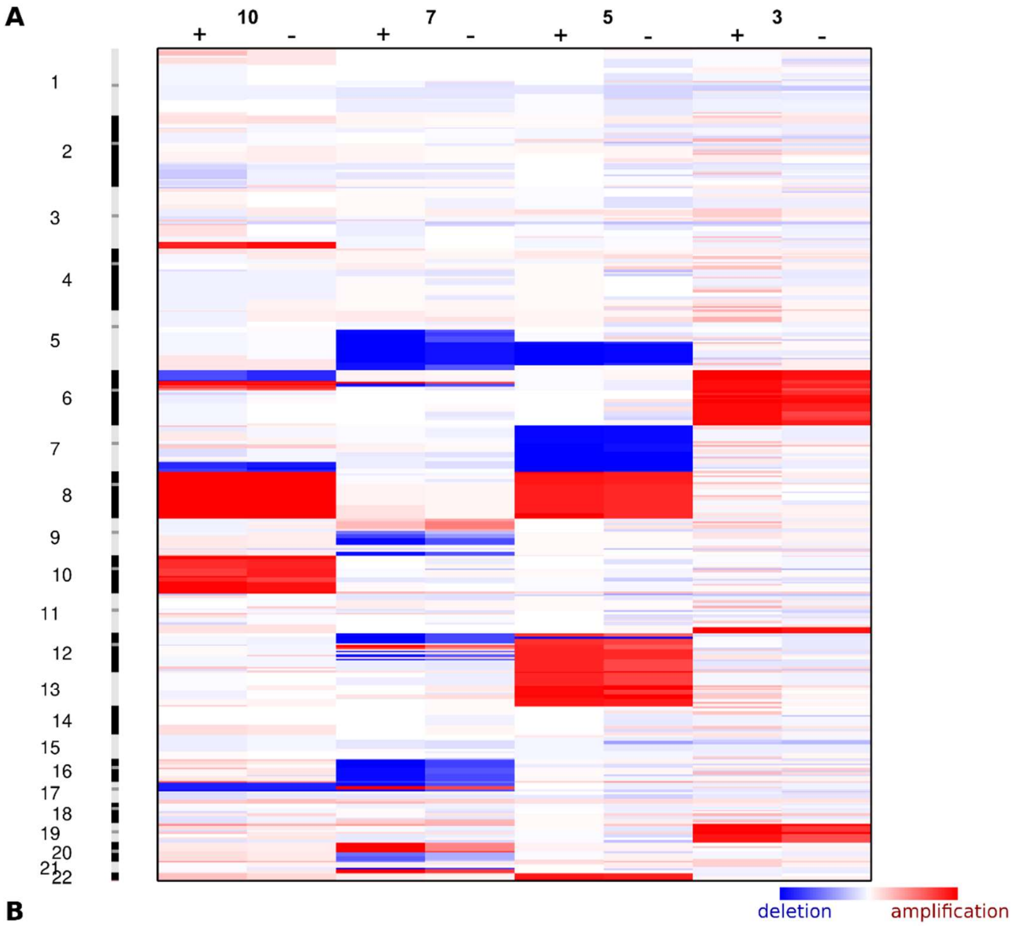

B

Patient	10		7		5		3		19	
CD34 fraction	+	-	+	-	+	-	+	-	+	-
TP53	0.95	0.8	0.9 0	0.5 0.2					N/A	0.77
NF1			0.87	0.46						
SLC22A1	0.56	0.53								
TYK2					0.48	0.49				
RET					0.51	0.47				
KRAS					0.22	0.24				
MAP3K1	0.61	0.47								
FLT3-TKD							0.5	0.48		
NCOR							0.56	0.48		
IDH2	0.5 0.3	0.4 0.4								
DNMT3A	0.55	0.51					0.48	0.5		
KDM6A										
APLF									0.44	0.57
WRN							0.46	0.48		
TDP1							0.52	0.55		

Figure S5. Copy number variation (CNV) and mutational profiles of the CD34+ and CD34- cellular fractions obtained by FACS for selected CK-AML patient samples. Five CK-AML samples were subjected to FACS to separate the CD34+ and CD34- cellular fractions, which were then sequenced separately. We observed no difference in the mutational spectrum and cytogenetic profiles of the two fractions. Variant allele frequencies of the mutated genes were almost identical, except for patient 7. **(A)** CNV profiles of the CD34+ and CD34- fractions. The CNV analysis was unsuccessful for the CD34+ fraction of patient 19 cells. **(B)** Oncoplot of mutational profiles of the CD34+ and CD34- fractions, with VAF information (not CNV-corrected). N/A – not available.

Figure S6

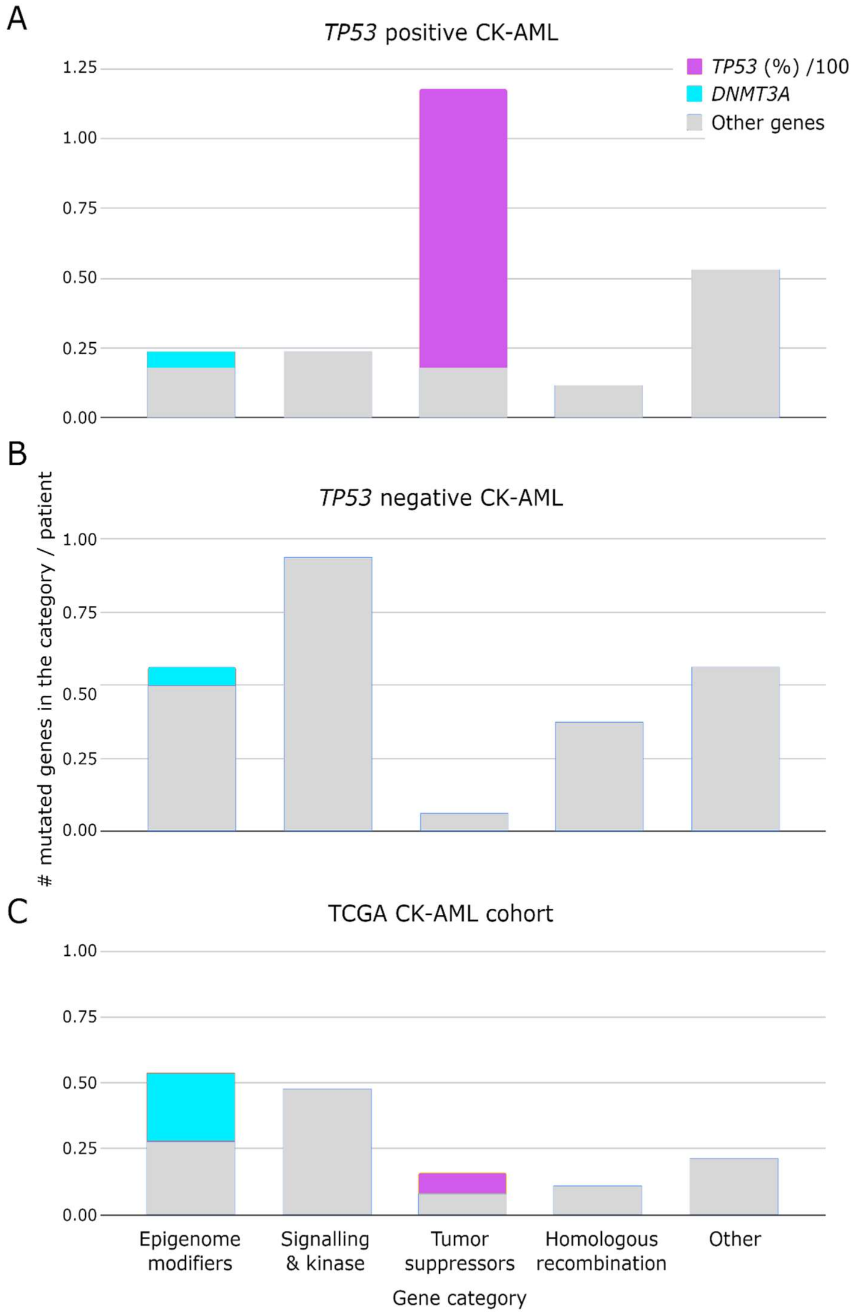

Figure S6. Mutational burden across gene categories for **(A)** *TP53* mutated patients in the current CK-AML cohort, **(B)** $TP53_{wt}$ patients in the current CK-AML cohort, and **(C)** all AML cases in The Cancer Genome Atlas (TCGA)[18] database.

Figure S7

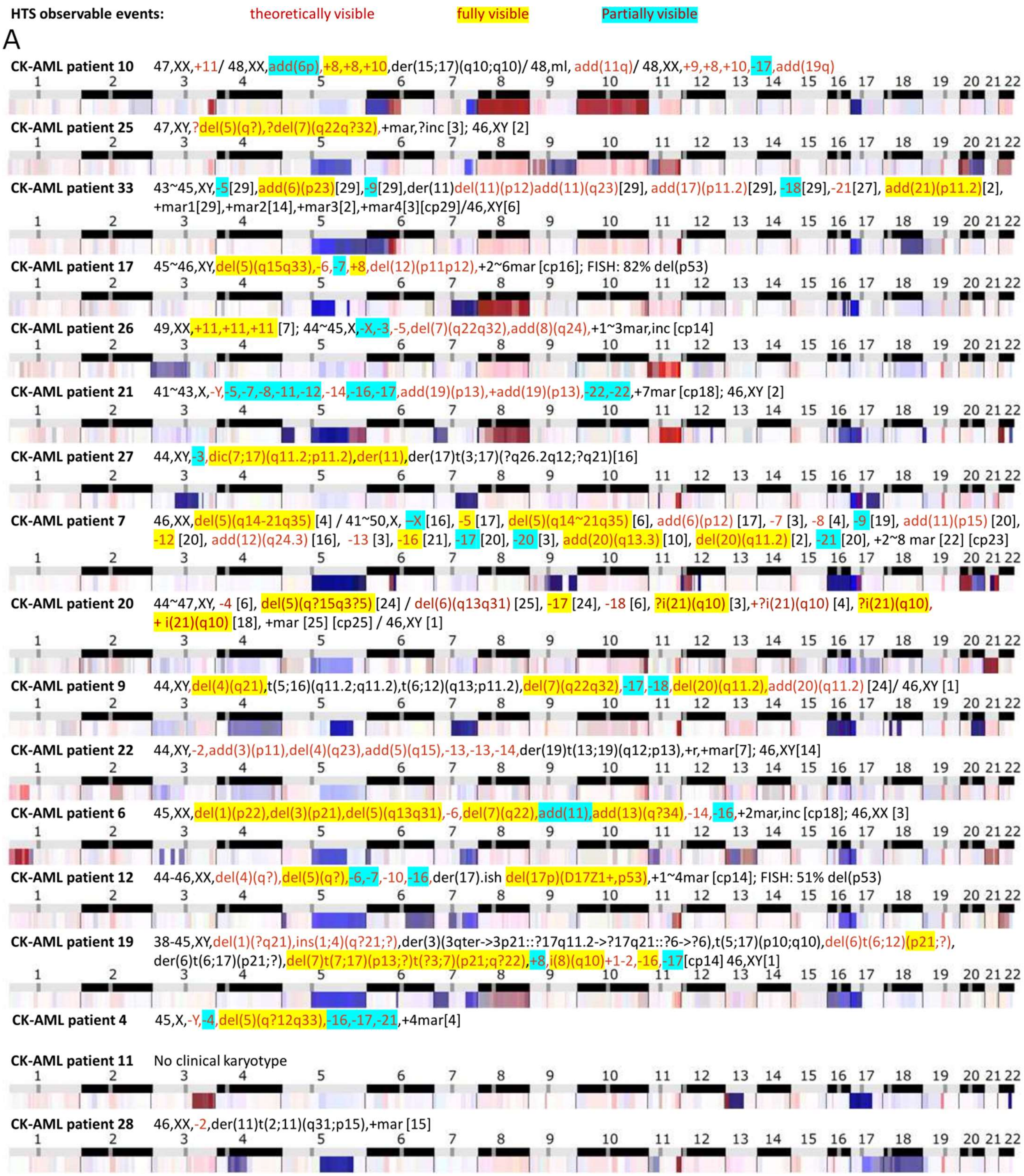

HTS observable events:
theoretically visible
fully visible
Partially visible
A
CK-AML patient 10
47,XX,+11/ 48,XX,add(6p),+8,+8,+10,der(15;17)(q10;q10)/ 48,ml, add(11q)/ 48,XX,+9,+8,+10,-17,add(19q)
1 2 3 4 5 6 7 8 9 10 11 12 13 14 15 16 17 18 19 20 21 22
CK-AML patient 25
47,XY,?del(5)(q?),?del(7)(q22q?32),+mar,?inc [3]; 46,XY [2]
CK-AML patient 33
43~45,XY,-5[29],add(6)(p23)[29],-9[29],der(11)del(11)(p12)add(11)(q23)[29], add(17)(p11.2)[29], -18[29],-21[27], add(21)(p11.2)[2], +mar1[29],+mar2[14],+mar3[2],+mar4[3][cp29]/46,XY[6]
CK-AML patient 17
45~46,XY,del(5)(q15q33),-6,-7,+8,del(12)(p11p12),+2~6mar [cp16]; FISH: 82% del(p53)
CK-AML patient 26
49,XX,+11,+11,+11 [7]; 44~45,X,-X,-3,-5,del(7)(q22q32),add(8)(q24),+1~3mar,inc [cp14]
CK-AML patient 21
41~43,X,-Y,-5,-7,-8,-11,-12,-14,-16,-17,add(19)(p13),+add(19)(p13),-22,-22,+7mar [cp18]; 46,XY [2]
CK-AML patient 27
44,XY,-3,dic(7;17)(q11.2;p11.2),der(11),der(17)t(3;17)(?q26.2q12;?q21)[16]
CK-AML patient 7
46,XX,del(5)(q14-21q35) [4] / 41~50,X, –X [16], -5 [17], del(5)(q14~21q35) [6], add(6)(p12) [17], -7 [3], -8 [4], -9 [19], add(11)(p15) [20], -12 [20], add(12)(q24.3) [16], -13 [3], -16 [21], -17 [20], -20 [3], add(20)(q13.3) [10], del(20)(q11.2) [2], -21 [20], +2~8 mar [22] [cp23]
CK-AML patient 20
44~47,XY, -4 [6], del(5)(q?15q3?5) [24] / del(6)(q13q31) [25], -17 [24], -18 [6], ?i(21)(q10) [3],+?i(21)(q10) [4], ?i(21)(q10), + i(21)(q10) [18], +mar [25] [cp25] / 46,XY [1]
CK-AML patient 9
44,XY,del(4)(q21),t(5;16)(q11.2;q11.2),t(6;12)(q13;p11.2),del(7)(q22q32),-17,-18,del(20)(q11.2),add(20)(q11.2) [24]/ 46,XY [1]
CK-AML patient 22
44,XY,-2,add(3)(p11),del(4)(q23),add(5)(q15),-13,-13,-14,der(19)t(13;19)(q12;p13),+r,+mar[7]; 46,XY[14]
CK-AML patient 6
45,XX,del(1)(p22),del(3)(p21),del(5)(q13q31),-6,del(7)(q22),add(11),add(13)(q?34),-14,-16,+2mar,inc [cp18]; 46,XX [3]
CK-AML patient 12
44-46,XX,del(4)(q?),del(5)(q?),-6,-7,-10,-16,der(17).ish del(17p)(D17Z1+,p53),+1~4mar [cp14]; FISH: 51% del(p53)
CK-AML patient 19
38-45,XY,del(1)(?q21),ins(1;4)(q?21;?),der(3)(3qter->3p21::?17q11.2->?17q21::?6->?6),t(5;17)(p10;q10),del(6)t(6;12)(p21;?), der(6)t(6;17)(p21;?),del(7)t(7;17)(p13;?)t(?3;7)(p21;q?22),+8,i(8)(q10)+1-2,-16,-17[cp14] 46,XY[1]
CK-AML patient 4
45,X,-Y,-4,del(5)(q?12q33),-16,-17,-21,+4mar[4]
CK-AML patient 11
No clinical karyotype
CK-AML patient 28
46,XX,-2,der(11)t(2;11)(q31;p15),+mar [15]

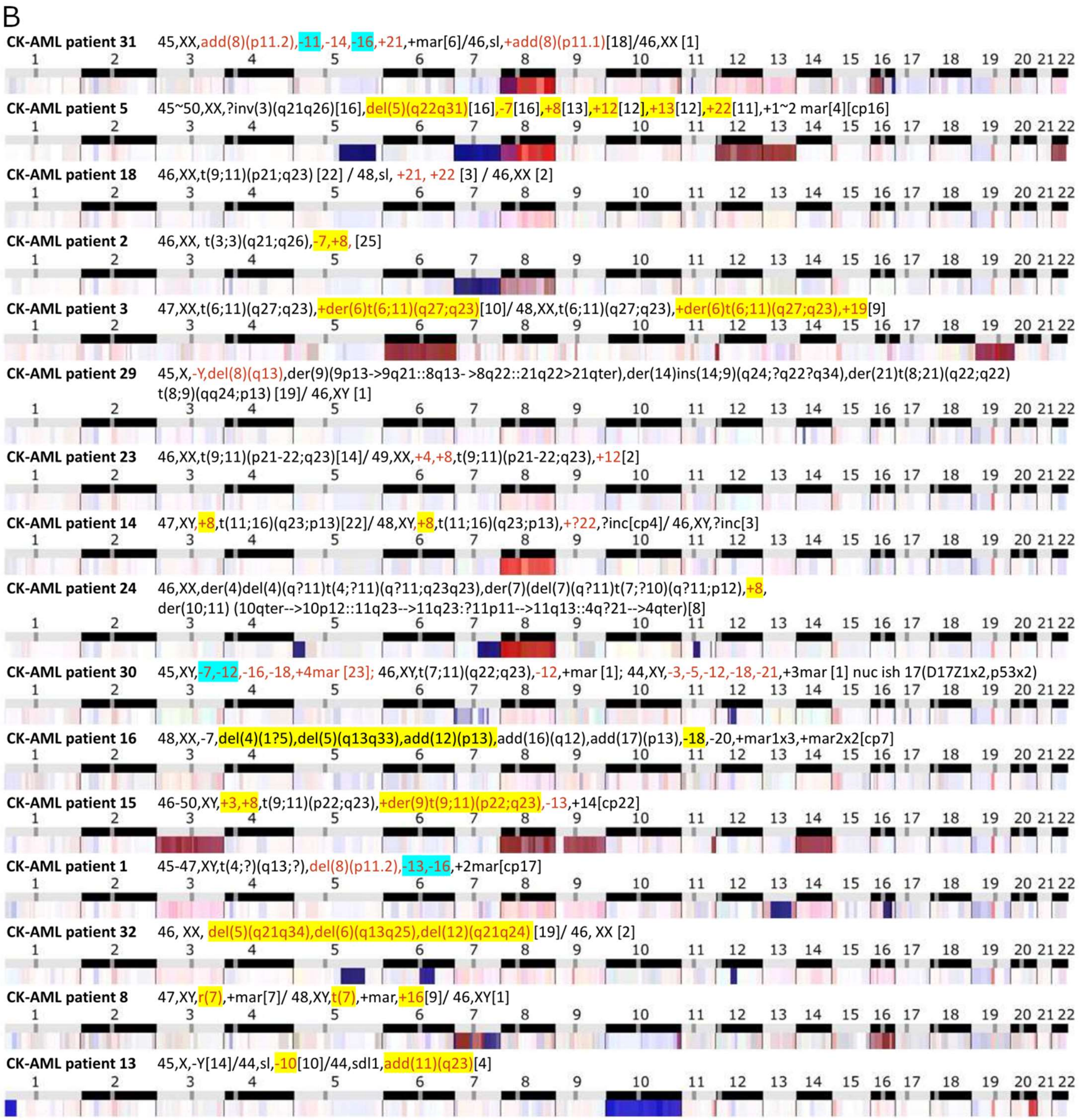

Figure S7. Comparison of NGS outcome for CNV analysis in our study with FISH-based karyotyping. (A) *TP53* mutated patients. **(B)** *TP53* wild-type patients. CNV profiles for each patient obtained from high-throughput NGS (HTS) were visualized using GISTIC; deletions are marked in blue and amplifications in red. The FISH-based karyotype for the respective patient is provided above. CD34+ fractions are shown for the FACS-sorted samples except for patient 19 (CD34- fraction shown). The analysis for patient 4 failed and is thus not shown.

Figure S8

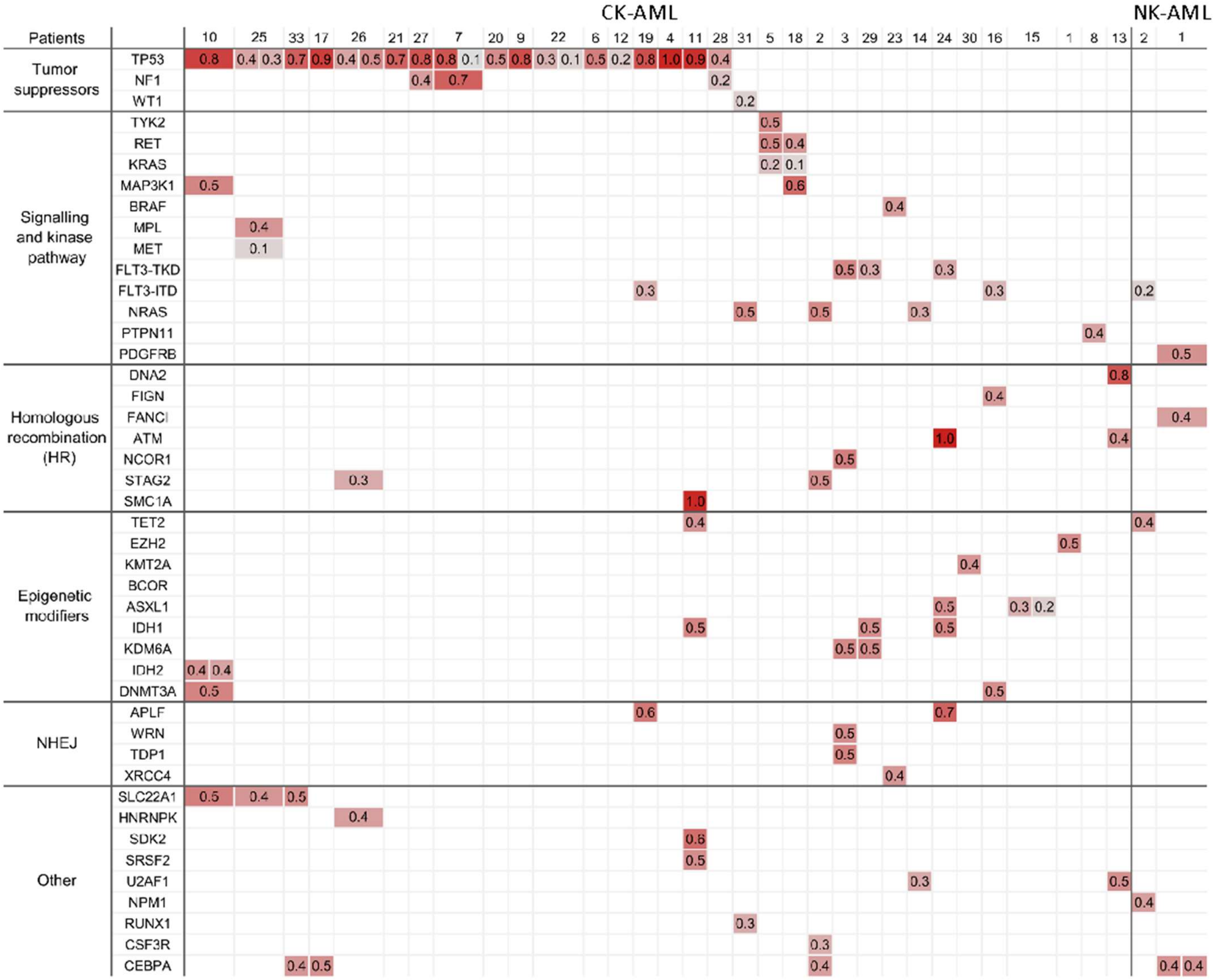

Figure S8. The oncoplot with VAF information, not corrected for the copy number value at the mutation site. For the patients sorted into the CD34+ and CD34- fractions, the weighted-average VAF values are presented.

Figure S9

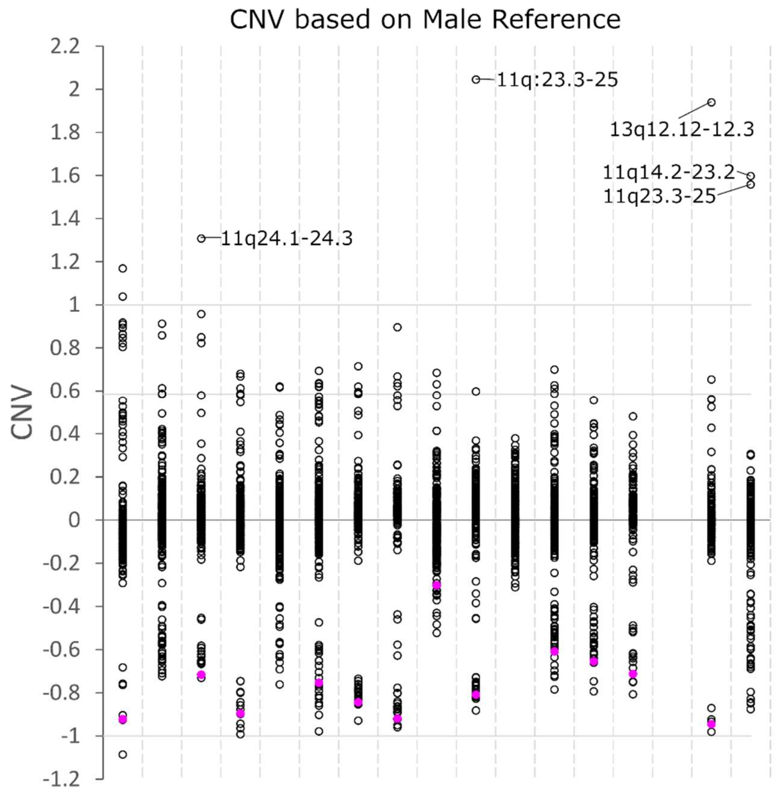

Figure S9. Copy number variation (*log₂(allele count)-1*) of genomic segments based on the male reference. *TP53*-containing segments are indicated in pink.

Figure S10

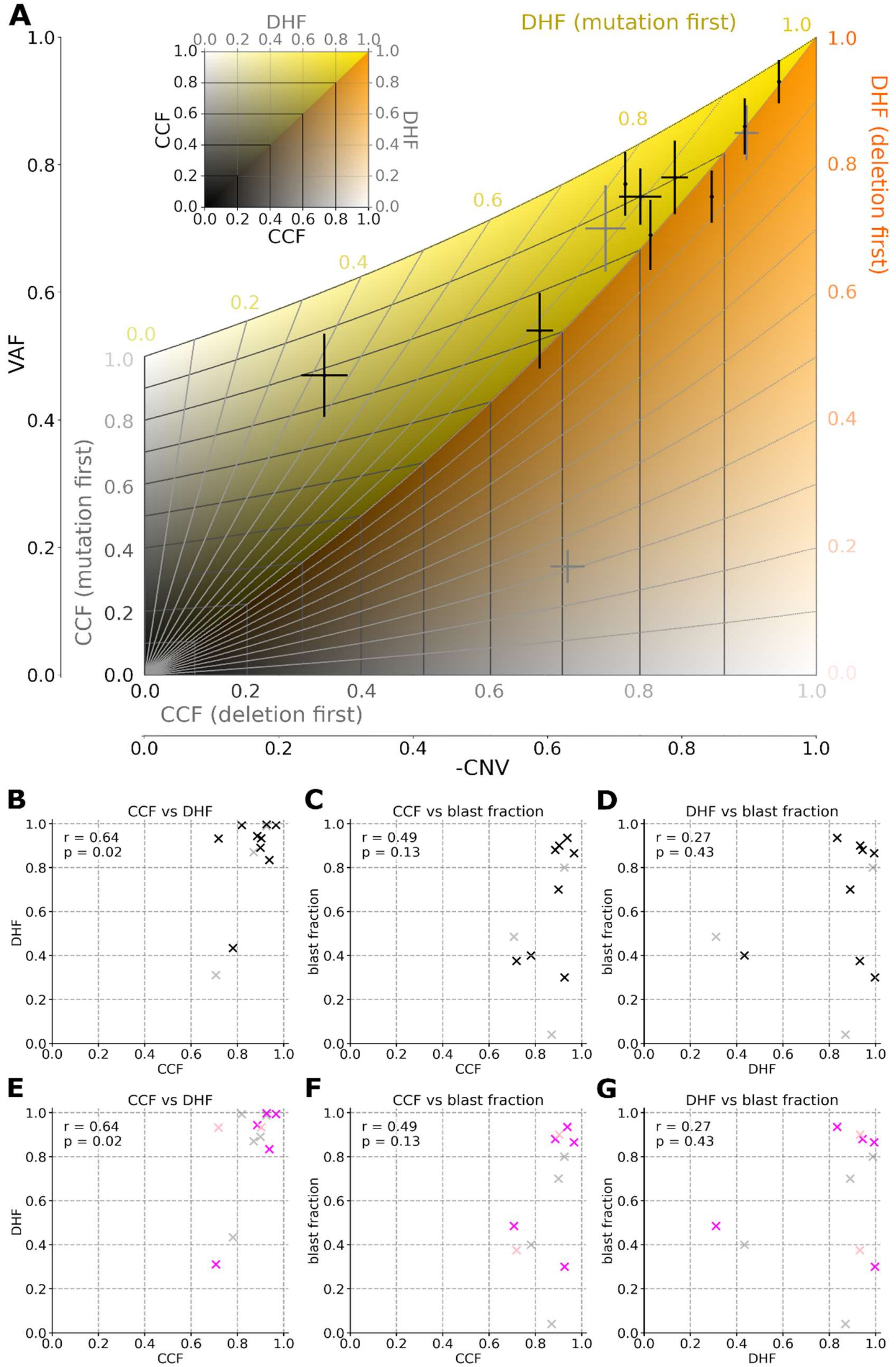

Figure S10. VAF and CNV analysis of the CK-AML *TP53*$_{\mathrm{mut}}$ patients based on CNV values from the default EXCAVATOR protocol (X chromosomes included). (A) *TP53* genotype for patients with *TP53* mutation and deletion. The vertical error bars correspond to the estimated standard deviations and the horizontal error bars reflect the differences in CNVs versus male and female references. **(B-G)** Correlations of CCF and DHF values estimated from the CNV and VAF values. **(B,E)** CCF versus DHF, **(C,F)** DHF versus blast fraction, **(D,G)** CCF versus blast fraction. The data points were colored gray in panels A-D for pretreated patients and dark/light pink in panels E-G for mutations with high/moderate DNE, respectively. Weighted averages were used for CD34 sorted samples.

Figure S11

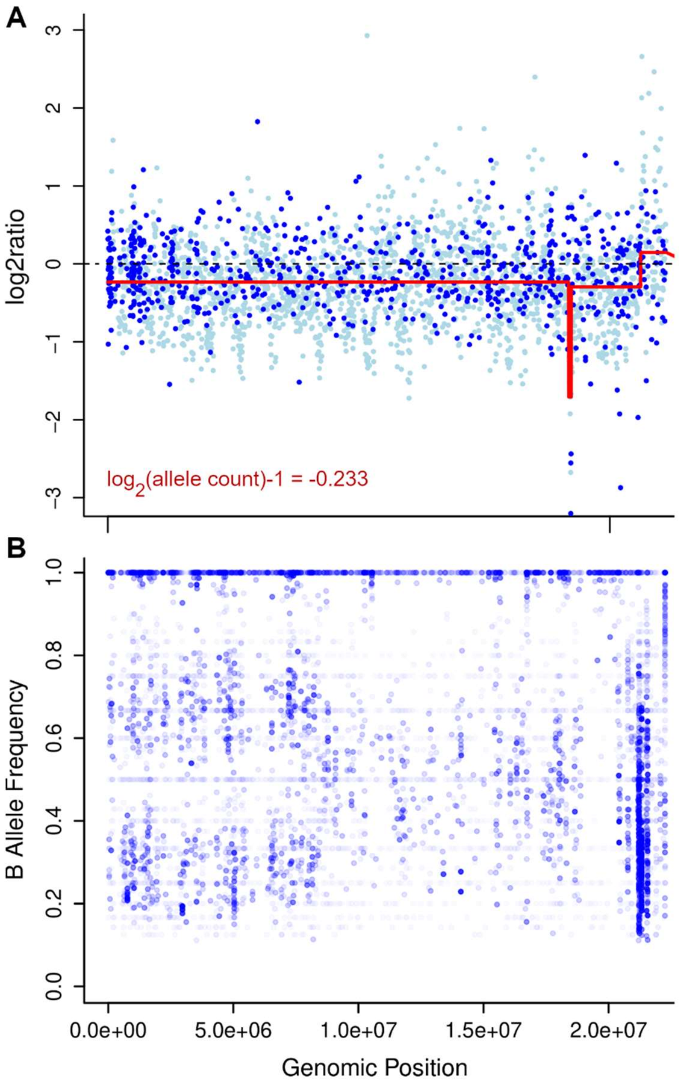

Figure S11. Copy number variation and B-allele frequency at the 17p chromosome arm (harboring the *TP53* locus) of patient 20. (A) CNV plot based on OneSeq Female Reference and **(B)** B-allele frequency plot. The segmentation carried out by EXCAVATOR for patient 20 in this region was inconsistent with the B-allele frequency analysis, which may explain the (possibly) smaller reported CNV value.

References

1 Tarabichi M, Salcedo A, Deshwar AG, Ni Leathlobhair M, Wintersinger J, Wedge DC *et al.* A practical guide to cancer subclonal reconstruction from DNA sequencing. *Nat Methods* 2021; **18**: 144–155.

2 Bochtler T, Granzow M, Stölzel F, Kunz C, Mohr B, Kartal-Kaess M *et al.* Marker chromosomes can arise from chromothripsis and predict adverse prognosis in acute myeloid leukemia. *Blood* 2017; **129**: 1333–1342.

3 Grimwade D, Hills RK, Moorman AV, Walker H, Chatters S, Goldstone AH *et al.* Refinement of cytogenetic classification in acute myeloid leukemia: determination of prognostic significance of rare recurring chromosomal abnormalities among 5876 younger adult patients treated in the United Kingdom Medical Research Council trials. *Blood* 2010; **116**: 354–365.

4 Rehm HL, Berg JS, Brooks LD, Bustamante CD, Evans JP, Landrum MJ *et al.* ClinGen — The Clinical Genome Resource. *N Engl J Med* 2015; **372**: 2235–2242.

5 Li H. Aligning sequence reads, clone sequences and assembly contigs with BWA-MEM. 2013. doi:10.48550/ARXIV.1303.3997.

6 Danecek P, Bonfield JK, Liddle J, Marshall J, Ohan V, Pollard MO *et al.* Twelve years of SAMtools and BCFtools. *GigaScience* 2021; **10**: giab008.

7 McLaren W, Gil L, Hunt SE, Riat HS, Ritchie GRS, Thormann A *et al.* The Ensembl Variant Effect Predictor. *Genome Biol* 2016; **17**: 122.

8 Sherry ST. dbSNP: the NCBI database of genetic variation. *Nucleic Acids Res* 2001; **29**: 308–311.

9 Landrum MJ, Chitipiralla S, Brown GR, Chen C, Gu B, Hart J *et al.* ClinVar: improvements to accessing data. *Nucleic Acids Res* 2020; **48**: D835–D844.

10 Cheng J, Novati G, Pan J, Bycroft C, Žemgulytė A, Applebaum T *et al.* Accurate proteome-wide missense variant effect prediction with AlphaMissense. *Science* 2023; **381**: eadg7492.

11 Brandes N, Goldman G, Wang CH, Ye CJ, Ntranos V. Genome-wide prediction of disease variant effects with a deep protein language model. *Nat Genet* 2023; **55**: 1512–1522.

12 Robinson JT, Thorvaldsdóttir H, Winckler W, Guttman M, Lander ES, Getz G *et al.* Integrative genomics viewer. *Nat Biotechnol* 2011; **29**: 24–26.

13 Lee S, Sun C-H, Jang H, Kim D, Yoon S-S, Koh Y *et al.* ITDetect: a method to detect internal tandem duplication of FMS-like tyrosine kinase (FLT3) from next-generation sequencing data with high sensitivity and clinical application. *BMC Bioinformatics* 2023; **24**: 62.

14 D'Aurizio R, Pippucci T, Tattini L, Giusti B, Pellegrini M, Magi A. Enhanced copy number variants detection from whole-exome sequencing data using EXCAVATOR2. *Nucleic Acids Res* 2016; **44**: e154.

15 Mermel CH, Schumacher SE, Hill B, Meyerson ML, Beroukhim R, Getz G. GISTIC2.0 facilitates sensitive and confident localization of the targets of focal somatic copy-number alteration in human cancers. *Genome Biol* 2011; **12**: R41.

16 Cerami E, Gao J, Dogrusoz U, Gross BE, Sumer SO, Aksoy BA *et al.* The cBio cancer genomics portal: an open platform for exploring multidimensional cancer genomics data. *Cancer Discov* 2012; **2**: 401–404.

17 de Andrade KC, Lee EE, Tookmanian EM, Kesserwan CA, Manfredi JJ, Hatton JN *et al.* The TP53 Database: transition from the International Agency for Research on Cancer to the US National Cancer Institute. *Cell Death Differ* 2022; **29**: 1071–1073.

18 The Cancer Genome Atlas Research Network. Genomic and Epigenomic Landscapes of Adult De Novo Acute Myeloid Leukemia. *N Engl J Med* 2013; **368**: 2059–2074.
