# Supplementary material for: Etiology of TP53 mutated complex karyotype acute myeloid leukemia
Source: Leukemia. 2025 Dec 19;40(2):444–8. doi: 10.1038/s41375-025-02835-9 (PMC12875860; doi:10.1038/s41375-025-02835-9)
Supplement: Supplementary file 2 — Supplementary code for Fig. 2D-F [file 41375_2025_2835_MOESM2_ESM.html]

CNV\_and\_VAF\_to\_CCF\_and\_DHF


## Jupyter notebook code for the generation of Fedenko et al. Fig. 3D-F¶

### The notebook requires an input a Tumor\_Fractions.tsv file with the following columns:¶

```
patient ID - patient identifier
VAF_CD34_w - variant allele frequency (VAF, weighted for CD34+ and CD34- cell fractions sequenced separately)
VAF_std_UB - the estimated upper boundary of VAF standard deviation
VAF_std_LB - the estimated upper boundary of VAF standard deviation
CNV_toFR_sm_w_EXCAVATOR_autosomes - copy number variation versus Female Reference from EXCAVATOR 
                                calculated based on autosomes only, 
                                smoothed, weighted for CD34+ and CD34- fractions)
CNV_toMR_sm_w_EXCAVATOR_autosomes - copy number variation versus Male Reference from EXCAVATOR, 
                                calculated based on autosomes only, 
                                smoothed, weighted for CD34+ and CD34- fractions)
CNV_toFR_sm_w_EXCAVATOR_default - copy number variation versus Female Reference from EXCAVATOR, 
                                smoothed, weighted for CD34+ and CD34- fractions)
CNV_toMR_sm_w_EXCAVATOR_default - copy number variation versus Male Reference from EXCAVATOR, 
                                smoothed, weighted for CD34+ and CD34- fractions)
TP53_ref_count - reference allele count at the TP53 locus
TP53_alt_count - alternative allele count at the TP53 locus
TP53_all_count - total allele count at the TP53 locus
blast_f - blast fraction (clinical information)
DNE - dominant negative effect of the mutation from IARC TP53 database: yes, moderate, undetermined [Y/M/ND]
pretreated - information about pretreatment [Y/N]
```

Columns 1 and 14-15 are expected in the text format, columns 2-13 in the numeric format

In [30]:

```
import warnings
warnings.filterwarnings("ignore")
import numpy as np
import pandas as pd
import matplotlib.pyplot as plt
import matplotlib.colors as mcolors
import matplotlib.gridspec as gridspec
import matplotlib.tri as tri
from scipy.optimize import fsolve
from scipy.stats import pearsonr
```

### Load data¶

In [6]:

```
vaf_cnv = pd.read_csv('Tumor_Fractions.tsv', sep='\t', dtype='object')
vaf_cnv.iloc[:, 1:12] = vaf_cnv.iloc[:, 1:12].apply(pd.to_numeric)
color_map = {'N': 'black', 'Y': 'silver'}
vaf_cnv['color'] = vaf_cnv['pretreated'].map(color_map)
vaf_cnv
```

Out[6]:

|  | patient ID | VAF\_CD34\_w | VAF\_std\_UB | VAF\_std\_LB | CNV\_toFR\_sm\_w\_EXCAVATOR\_autosomes | CNV\_toMR\_sm\_w\_EXCAVATOR\_autosomes | CNV\_toFR\_sm\_w\_EXCAVATOR\_default | CNV\_toMR\_sm\_w\_EXCAVATOR\_default | TP53\_ref\_count | TP53\_alt\_count | TP53\_all\_count | blast\_f | DNE | pretreated | color |
| --- | --- | --- | --- | --- | --- | --- | --- | --- | --- | --- | --- | --- | --- | --- | --- |
| 0 | p10\_av | 0.85 | 0.893184 | 0.806816 | -0.872116 | -0.888034 | -0.878321 | -0.913303 | 1 | 31 | 32 | 80.0 | ND | Y | silver |
| 1 | p33 | 0.7 | 0.767843 | 0.632157 | -0.693693 | -0.714053 | -0.656 | -0.716 | 14 | 32 | 46 | 4.0 | ND | Y | silver |
| 2 | p17 | 0.86 | 0.904087 | 0.815913 | -0.892778 | -0.893015 | -0.89055 | -0.896 | 9 | 54 | 63 | 30.0 | Y | N | black |
| 3 | p21 | 0.69 | 0.744881 | 0.635119 | -0.765659 | -0.756455 | -0.752 | -0.754 | 22 | 49 | 71 | NaN | ND | N | black |
| 4 | p27 | 0.75 | 0.79079 | 0.70921 | -0.854073 | -0.838165 | -0.844 | -0.844 | 29 | 85 | 114 | 88.0 | Y | N | black |
| 5 | p7\_av | 0.75 | 0.794298 | 0.705702 | -0.716124 | -0.75842 | -0.706041 | -0.769482 | 2 | 22 | 24 | 70.0 | ND | N | black |
| 6 | p9 | 0.78 | 0.837593 | 0.722407 | -0.786982 | -0.802618 | -0.769 | -0.809 | 11 | 40 | 51 | 90.0 | M | N | black |
| 7 | p6 | 0.54 | 0.599541 | 0.480459 | -0.561814 | -0.590538 | -0.568 | -0.608 | 32 | 38 | 70 | 37.5 | M | N | black |
| 8 | p19\_CD34neg | 0.77 | 0.820189 | 0.719811 | -0.719446 | -0.712572 | -0.718 | -0.713 | 16 | 54 | 70 | 93.5 | Y | N | black |
| 9 | p12 | 0.17 | 0.196052 | 0.143948 | -0.598565 | -0.636856 | -0.604 | -0.655 | 172 | 35 | 207 | 48.5 | Y | Y | silver |
| 10 | p11 | 0.93 | 0.963834 | 0.896166 | -0.949389 | -0.944376 | -0.943 | -0.945 | 4 | 53 | 57 | 86.5 | Y | N | black |
| 11 | p20 | 0.47 | 0.535497 | 0.404503 | -0.290592 | -0.30412 | -0.233 | -0.302 | 31 | 27 | 58 | 40.0 | ND | N | black |

### Calculate the -CNV and VAF given CCF and DHF for two alternative scenarios¶

#### Scenario mutation first¶

In [7]:

```
def mut_first(CCF, DHF):
    # return a vector of the negative CNV and VAF
    all_alleles=(1-CCF)*2+CCF*(1-DHF)*2+CCF*DHF*1
    mut_alleles=CCF
    CNV=np.log(all_alleles)/np.log(2)-1
    neg_CNV=-CNV
    VAF=mut_alleles/all_alleles
    return [neg_CNV,VAF,CCF,DHF]
```

In [8]:

```
def mut_first_np(CCF_DHF):
    # takes a numpy array
    CCF=CCF_DHF[0]
    DHF=CCF_DHF[1]
    return np.array(mut_first(CCF, DHF)[:2])  # Return first two elements
```

#### Scenario deletion first¶

In [9]:

```
def del_first(CCF,DHF):
    # return a vector of the negative CNV and VAF
    all_alleles=(1-CCF)*2+CCF*(1-DHF)*1+CCF*DHF*1
    mut_alleles=CCF*DHF
    CNV=np.log(all_alleles)/np.log(2)-1
    neg_CNV=-CNV
    VAF=mut_alleles/all_alleles
    return [neg_CNV,VAF,CCF,DHF]
```

In [10]:

```
def del_first_np(CCF_DHF):
    # takes a numpy array
    CCF=CCF_DHF[0]
    DHF=CCF_DHF[1]
    return np.array(del_first(CCF, DHF)[:2])  # Return first two elements
```

#### Calculate the vectors for the matplotlib plot¶

In [11]:

```
def calculate_vectors_for_plot(CNV_VAF_calculator):
    #
    # returns a dictionary with the vectors for the plot 
    # {"area_x": **, "area_y": **, "CCF_x": ***, "CCF_y": ***; "DHF_x": *** "DHF_y": ***}
    #
    # fill the area
    #
    CCF_vec=np.linspace(0,1,1000)
    DHF_vec=np.linspace(0,1,1000)
    coordinate_pairs=[]
    for CCF in CCF_vec:
        for DHF in DHF_vec:
            coordinate_pairs.append(CNV_VAF_calculator(CCF,DHF))
    coordinate_pairs_array=np.array(coordinate_pairs)
    coordinate_pairs_array_swap=np.swapaxes(coordinate_pairs_array,1,0)
    #
    # calculate the CCF lines
    #
    CCF_lines_CCF_vec=np.linspace(0,1,11)
    CCF_lines_DHF_vec=np.linspace(0,1,500)
    CCF_lines_coordinate_pairs=[]
    for CCF in CCF_lines_CCF_vec:
        for DHF in CCF_lines_DHF_vec:
            CCF_lines_coordinate_pairs.append(CNV_VAF_calculator(CCF,DHF))
    CCF_lines_coordinate_pairs_array=np.array(CCF_lines_coordinate_pairs)
    CCF_lines_coordinate_pairs_array_swap=np.swapaxes(CCF_lines_coordinate_pairs_array,1,0)
    #
    # calculate the DHF lines
    #
    DHF_lines_CCF_vec=np.linspace(0,1,500)
    DHF_lines_DHF_vec=np.linspace(0,1,11)
    DHF_lines_coordinate_pairs=[]
    for CCF in DHF_lines_CCF_vec:
        for DHF in DHF_lines_DHF_vec:
            DHF_lines_coordinate_pairs.append(CNV_VAF_calculator(CCF,DHF))
    DHF_lines_coordinate_pairs_array=np.array(DHF_lines_coordinate_pairs)
    DHF_lines_coordinate_pairs_array_swap=np.swapaxes(DHF_lines_coordinate_pairs_array,1,0)
    #
    return {"area_x": coordinate_pairs_array_swap[0], 
            "area_y": coordinate_pairs_array_swap[1],
            "CCF": coordinate_pairs_array_swap[2],
            "DHF": coordinate_pairs_array_swap[3],
            "CCF_x": CCF_lines_coordinate_pairs_array_swap[0], 
            "CCF_y": CCF_lines_coordinate_pairs_array_swap[1],
            "DHF_x": DHF_lines_coordinate_pairs_array_swap[0], 
            "DHF_y": DHF_lines_coordinate_pairs_array_swap[1]}
```

In [12]:

```
my_mut_dict=calculate_vectors_for_plot(mut_first)
```

In [13]:

```
my_del_dict=calculate_vectors_for_plot(del_first)
```

#### Plot the -CNV versus VAF figure¶

In [24]:

```
plt.figure(figsize=(16, 16))
#
# plot the yellow, mutation first part
#
hsv_colors = np.zeros((1000000, 3))  # Initialize array for HSV colors
hsv_colors[:, 0] = 0.15  # Hue (color)
hsv_colors[:, 1] = my_mut_dict["DHF"]  # Saturation (full color saturation)
hsv_colors[:, 2] = my_mut_dict["CCF"]  # Value (brightness)
rgb_colors = mcolors.hsv_to_rgb(hsv_colors)
plt.scatter(my_mut_dict["area_x"], my_mut_dict["area_y"], color=rgb_colors, s=5, marker='.')
#
# plot the orange, deletion first part
#
hsv_colors = np.zeros((1000000, 3))  # Initialize array for HSV colors
hsv_colors[:, 0] = 0.1  # Hue (color)
hsv_colors[:, 1] = my_del_dict["DHF"]  # Saturation (full color saturation)
hsv_colors[:, 2] = my_del_dict["CCF"]  # Value (brightness)
rgb_colors = mcolors.hsv_to_rgb(hsv_colors)
plt.scatter(my_del_dict["area_x"],my_del_dict["area_y"],color=rgb_colors,s=5, marker='.')
#
# plot the isochores
#
plt.scatter(my_mut_dict["CCF_x"], my_mut_dict["CCF_y"], color=(0.3,0.3,0.3), s=0.6, marker=',')
plt.scatter(my_mut_dict["DHF_x"], my_mut_dict["DHF_y"], color=(0.6,0.6,0.6), s=0.3, marker=',')
#
plt.scatter(my_del_dict["CCF_x"],my_del_dict["CCF_y"],color=(0.3,0.3,0.3),s=0.6, marker=',')
plt.scatter(my_del_dict["DHF_x"],my_del_dict["DHF_y"],color=(0.6,0.6,0.6),s=0.3, marker=',')
#
# plot the data (horizontal CNV bars based on FR and MR CNV values)
#
for _, row in vaf_cnv.iterrows():
    plt.hlines(
        xmin=-row['CNV_toFR_sm_w_EXCAVATOR_autosomes'], 
        xmax=-row['CNV_toMR_sm_w_EXCAVATOR_autosomes'],
        y=row['VAF_CD34_w'],
        color=row['color'],  
        linewidth=3)
    plt.errorbar(
        x=(-row['CNV_toFR_sm_w_EXCAVATOR_autosomes'] - row['CNV_toMR_sm_w_EXCAVATOR_autosomes']) / 2,
        y=row['VAF_CD34_w'],
        yerr=[[row['VAF_CD34_w'] - row['VAF_std_LB']],  # Lower error
              [row['VAF_std_UB'] - row['VAF_CD34_w']]],  # Upper error
        fmt='None',
        color=row['color'],
        capsize=0,
        linewidth=3)
    plt.scatter(
        (-row['CNV_toFR_sm_w_EXCAVATOR_autosomes']-row['CNV_toMR_sm_w_EXCAVATOR_autosomes'])/2,
        row['VAF_CD34_w'],
        color=row['color'],
        marker='o',
        s=20)
    
plt.xlim(0, 1)
plt.ylim(0, 1)
plt.xlabel("-CNV", fontsize=24)
plt.ylabel("VAF", fontsize=24)
plt.xticks(fontsize=24)
plt.yticks(fontsize=24)

plt.savefig("data.tif", dpi=200)
plt.show()
plt.close()
```

#### Plot the upper-left triangle for the legend¶

In [15]:

```
plt.figure(figsize=(3, 3))
#
plt.title("DHF vs CCF", fontsize=20)
hsv_colors = np.zeros((1000000, 3))  # Initialize array for HSV colors
hsv_colors[:, 0] = 0.15  # Hue (color)
hsv_colors[:, 1] = my_mut_dict["DHF"]  # Saturation (full color saturation)
hsv_colors[:, 2] = my_mut_dict["CCF"]  # Value (brightness)
rgb_colors = mcolors.hsv_to_rgb(hsv_colors)
plt.xlim(0, 1)
plt.ylim(0, 1)
plt.xticks(fontsize=16)
plt.yticks(fontsize=16)
#plt.xlabel("DHF", fontsize=12)
#plt.ylabel("CCF", fontsize=12)
plt.grid(color='gray', linestyle='--', linewidth=0.5, alpha=0.7)
plt.scatter(my_mut_dict["DHF"], my_mut_dict["CCF"], color=rgb_colors, s=5, marker='.')

plt.savefig("legend_yellow.tif", dpi=200, bbox_inches="tight")
plt.show()
plt.close()
```

#### Plot the lower-right triangle for the legend¶

In [16]:

```
plt.figure(figsize=(3, 3))
plt.title("DHF vs CCF", fontsize=20)
hsv_colors = np.zeros((1000000, 3))  # Initialize array for HSV colors
hsv_colors[:, 0] = 0.1  # Hue (color)
hsv_colors[:, 1] = my_del_dict["DHF"]  # Saturation (full color saturation)
hsv_colors[:, 2] = my_del_dict["CCF"]  # Value (brightness)
rgb_colors = mcolors.hsv_to_rgb(hsv_colors)
plt.xlim(0, 1)
plt.ylim(0, 1)
plt.xticks(fontsize=16)
plt.yticks(fontsize=16)
plt.grid(color='gray', linestyle='--', linewidth=0.5, alpha=0.7)
#plt.xlabel("DHF", fontsize=12)
#plt.ylabel("CCF", fontsize=12)
plt.scatter(my_del_dict["DHF"],my_del_dict["CCF"],color=rgb_colors,s=5, marker='.')

plt.savefig("legend_orange.tif", dpi=200, bbox_inches="tight")
plt.show()
plt.close()
```

### Perform the reverse calculation, i.e. deduce CCF and DHF for experimentally determined -CNV and VAF¶

for the midline with rest WT/WT, the midline has the functional form $$VAF=2^{-CNV}-1$$

for the midline with rest WT/mut, the top line has the functional form $$VAF=1/2 \cdot 2^{-CNV}$$

In [17]:

```
def invert(function, target ,start):
    def helper(z):
        return function(z)-target
    solution=fsolve(helper,start)
    return solution
```

In [18]:

```
def get_CCF_and_DHF(neg_CNV,VAF):
    # 
    # determine the regime in the graph
    #
    if (VAF > 1 or VAF < 0 or neg_CNV  > 1 or neg_CNV < 0):
        #
        # invalid input
        #
        exit
    elif (VAF > 0.5*(2**neg_CNV)):
        #
        # in the white impossible regime 
        #
        exit
    elif (VAF > (2**neg_CNV)-1):
        #
        # in the yellow mutation first regime
        #
        result = invert(mut_first_np,np.array([neg_CNV, VAF]),np.array([0.8,0.8]))
        return (result[0], result[1], result[0]*result[1])
    else:
        #
        # in the orange deletion first regime 
        #
        result = invert(del_first_np,np.array([neg_CNV, VAF]),np.array([0.8,0.4]))
        return (result[0], result[1], result[0])
```

In [25]:

```
results = []

for _, row in vaf_cnv.iterrows():
    x = (-row['CNV_toFR_sm_w_EXCAVATOR_autosomes'] - row['CNV_toMR_sm_w_EXCAVATOR_autosomes']) / 2
    y = row['VAF_CD34_w']
    output = get_CCF_and_DHF(x, y)  # Function returning CCF and DHF

    results.append({
        'patient': row['patient ID'],
        'blast_f': row['blast_f'],
        'color': row['color'],
        'x': x,
        'y': y,
        'CCF': output[0],
        'DHF': output[1],
        'deletionF' : output[2]
    })

df_results = pd.DataFrame(results)
df_results.to_csv("output.tsv", sep="\t", index=False)
```

In [26]:

```
df_results
```

Out[26]:

|  | patient | blast\_f | color | x | y | CCF | DHF | deletionF |
| --- | --- | --- | --- | --- | --- | --- | --- | --- |
| 0 | p10\_av | 80.0 | silver | 0.880075 | 0.85 | 0.923677 | 0.988789 | 0.913322 |
| 1 | p33 | 4.0 | silver | 0.703873 | 0.70 | 0.859491 | 0.898388 | 0.772156 |
| 2 | p17 | 30.0 | black | 0.892897 | 0.86 | 0.926275 | 0.996396 | 0.922936 |
| 3 | p21 | NaN | black | 0.761057 | 0.69 | 0.819872 | 0.993189 | 0.819872 |
| 4 | p27 | 88.0 | black | 0.846119 | 0.75 | 0.887442 | 0.940252 | 0.887442 |
| 5 | p7\_av | 70.0 | black | 0.737272 | 0.75 | 0.899809 | 0.889361 | 0.800255 |
| 6 | p9 | 90.0 | black | 0.794800 | 0.78 | 0.899220 | 0.942099 | 0.847154 |
| 7 | p6 | 37.5 | black | 0.576176 | 0.54 | 0.724398 | 0.909060 | 0.658521 |
| 8 | p19\_CD34neg | 93.5 | black | 0.716009 | 0.77 | 0.937520 | 0.834587 | 0.782442 |
| 9 | p12 | 48.5 | silver | 0.617711 | 0.17 | 0.696591 | 0.318091 | 0.696591 |
| 10 | p11 | 86.5 | black | 0.946883 | 0.93 | 0.964879 | 0.997530 | 0.962496 |
| 11 | p20 | 40.0 | black | 0.297356 | 0.47 | 0.764918 | 0.487001 | 0.372516 |

#### Plot the CCF vs DHF graph¶

In [84]:

```
# Set up the figure and gridspec layout
fig = plt.figure(figsize=(8, 8))
gs = gridspec.GridSpec(2, 2, width_ratios=[19, 1], height_ratios=[1, 19],
                       wspace=0.05, hspace=0.05)

# Axes
ax_scatter = fig.add_subplot(gs[1, 0])
ax_histx = fig.add_subplot(gs[0, 0], sharex=ax_scatter)
ax_histy = fig.add_subplot(gs[1, 1], sharey=ax_scatter)

# Data
x_data = df_results["CCF"]
y_data = df_results["DHF"]
colors = df_results["color"]

# Correlation
corr, p = pearsonr(x_data, y_data)

# Regression line
slope, intercept = np.polyfit(x_data, y_data, 1)
x_vals = np.linspace(x_data.min(), x_data.max(), 100)
y_vals = slope * x_vals + intercept

# Compute descriptive statistics
mean_x = x_data.mean()
median_x = x_data.median()
iqr_x = x_data.quantile(0.75) - x_data.quantile(0.25)

mean_y = y_data.mean()
median_y = y_data.median()
iqr_y = y_data.quantile(0.75) - y_data.quantile(0.25)

# Main scatter plot
ax_scatter.scatter(x_data, y_data, color=colors, s=200, marker='x', linewidths=3)
ax_scatter.set_xlim(0, 1.02)
ax_scatter.set_ylim(0, 1.02)
ax_scatter.set_xlabel("CCF", fontsize=24)
ax_scatter.set_ylabel("DHF", fontsize=24)
ax_scatter.grid(color='gray', linestyle='--', linewidth=2, alpha=0.7)
ax_scatter.tick_params(axis='both', width=2, length=6, labelsize=20)
ax_scatter.spines["top"].set_visible(False)
ax_scatter.spines["right"].set_visible(False)
ax_scatter.spines["bottom"].set_linewidth(2)
ax_scatter.spines["left"].set_linewidth(2)

stats_text_x = (
    f"  mean = {mean_x:.2f}\n"
    f"  median = {median_x:.2f}\n"
    f"  IQR = {iqr_x:.2f}\n"
)
plt.text(-4, 1.05, stats_text_x, fontsize=18,
         bbox=dict(facecolor="white", alpha=0, edgecolor="none"))
stats_text_y = (
    f"  mean = {mean_y:.2f}\n"
    f"  median = {median_y:.2f}\n"
    f"  IQR = {iqr_y:.2f}"
)
plt.text(1, 0.85, stats_text_y, fontsize=18,
         bbox=dict(facecolor="white", alpha=0, edgecolor="none"))

# Add correlation text
ax_scatter.text(0.05, 0.85, f"r = {corr:.2f}\np = {p:.2f}",
                fontsize=20, bbox=dict(facecolor="white", alpha=0.6, edgecolor="none"))
# Top boxplot (X-axis)
box_x = ax_histx.boxplot(x_data, vert=False, widths=0.5, patch_artist=False)
for element in ['boxes', 'whiskers', 'caps', 'medians']:
    for item in box_x[element]:
        item.set_linewidth(2.5)  # match scatter plot linewidth
ax_histx.axis("off")

# Right boxplot (Y-axis)
box_y = ax_histy.boxplot(y_data, vert=True, widths=0.5, patch_artist=False)
for element in ['boxes', 'whiskers', 'caps', 'medians']:
    for item in box_y[element]:
        item.set_linewidth(2.5)
ax_histy.axis("off")

# Title
fig.suptitle("CCF vs DHF", fontsize=26, x=0.2)

# Save and show
plt.savefig("ccf_dhf_with_marginal_boxplots.tif", dpi=200, bbox_inches="tight", facecolor="white")
plt.show()
plt.close()
```

#### Plot the DHF vs blast fraction graph¶

In [82]:

```
# Set up the figure and gridspec layout
fig = plt.figure(figsize=(8, 8))
gs = gridspec.GridSpec(2, 2, width_ratios=[19, 1], height_ratios=[1, 19],
                       wspace=0.05, hspace=0.05)

# Axes
ax_scatter = fig.add_subplot(gs[1, 0])
ax_histx = fig.add_subplot(gs[0, 0], sharex=ax_scatter)
ax_histy = fig.add_subplot(gs[1, 1], sharey=ax_scatter)

# Data
df_results = df_results.dropna(subset=["blast_f"])
x_data = df_results["DHF"]
y_data = df_results["blast_f"]/100
colors = df_results["color"]

# Correlation
corr, p = pearsonr(x_data, y_data)

# Regression line
slope, intercept = np.polyfit(x_data, y_data, 1)
x_vals = np.linspace(x_data.min(), x_data.max(), 100)
y_vals = slope * x_vals + intercept

# Compute descriptive statistics
mean_x = x_data.mean()
median_x = x_data.median()
iqr_x = x_data.quantile(0.75) - x_data.quantile(0.25)

mean_y = y_data.mean()
median_y = y_data.median()
iqr_y = y_data.quantile(0.75) - y_data.quantile(0.25)

# Main scatter plot
ax_scatter.scatter(x_data, y_data, color=colors, s=200, marker='x', linewidths=3)
ax_scatter.set_xlim(0, 1.02)
ax_scatter.set_ylim(0, 1.02)
ax_scatter.set_xlabel("DHF", fontsize=24)
ax_scatter.set_ylabel("blast fraction", fontsize=24)
ax_scatter.grid(color='gray', linestyle='--', linewidth=2, alpha=0.7)
ax_scatter.tick_params(axis='both', width=2, length=6, labelsize=20)
ax_scatter.spines["top"].set_visible(False)
ax_scatter.spines["right"].set_visible(False)
ax_scatter.spines["bottom"].set_linewidth(2)
ax_scatter.spines["left"].set_linewidth(2)

stats_text_x = (
    f"  mean = {mean_x:.2f}\n"
    f"  median = {median_x:.2f}\n"
    f"  IQR = {iqr_x:.2f}\n"
)
plt.text(-4, 1.05, stats_text_x, fontsize=18,
         bbox=dict(facecolor="white", alpha=0, edgecolor="none"))
stats_text_y = (
    f"  mean = {mean_y:.2f}\n"
    f"  median = {median_y:.2f}\n"
    f"  IQR = {iqr_y:.2f}"
)
plt.text(1, 0.85, stats_text_y, fontsize=18,
         bbox=dict(facecolor="white", alpha=0, edgecolor="none"))

# Add correlation text
ax_scatter.text(0.05, 0.85, f"r = {corr:.2f}\np = {p:.2f}",
                fontsize=20, bbox=dict(facecolor="white", alpha=0.6, edgecolor="none"))
# Top boxplot (X-axis)
box_x = ax_histx.boxplot(x_data, vert=False, widths=0.5, patch_artist=False)
for element in ['boxes', 'whiskers', 'caps', 'medians']:
    for item in box_x[element]:
        item.set_linewidth(2.5)  # match scatter plot linewidth
ax_histx.axis("off")

# Right boxplot (Y-axis)
box_y = ax_histy.boxplot(y_data, vert=True, widths=0.5, patch_artist=False)
for element in ['boxes', 'whiskers', 'caps', 'medians']:
    for item in box_y[element]:
        item.set_linewidth(2.5)
ax_histy.axis("off")

# Title
fig.suptitle("DHF vs blast fraction", fontsize=26, x=0.3)

# Save and show
plt.savefig("dhf_blast_with_marginal_boxplots.tif", dpi=200, bbox_inches="tight", facecolor="white")
plt.show()
plt.close()
```

#### Plot the CCF vs blast graph¶

In [85]:

```
# Set up the figure and gridspec layout
fig = plt.figure(figsize=(8, 8))
gs = gridspec.GridSpec(2, 2, width_ratios=[19, 1], height_ratios=[1, 19],
                       wspace=0.05, hspace=0.05)

# Axes
ax_scatter = fig.add_subplot(gs[1, 0])
ax_histx = fig.add_subplot(gs[0, 0], sharex=ax_scatter)
ax_histy = fig.add_subplot(gs[1, 1], sharey=ax_scatter)

# Data
df_results = df_results.dropna(subset=["blast_f"])
x_data = df_results["CCF"]
y_data = df_results["blast_f"]/100
colors = df_results["color"]

# Correlation
corr, p = pearsonr(x_data, y_data)

# Regression line
slope, intercept = np.polyfit(x_data, y_data, 1)
x_vals = np.linspace(x_data.min(), x_data.max(), 100)
y_vals = slope * x_vals + intercept

# Compute descriptive statistics
mean_x = x_data.mean()
median_x = x_data.median()
iqr_x = x_data.quantile(0.75) - x_data.quantile(0.25)

mean_y = y_data.mean()
median_y = y_data.median()
iqr_y = y_data.quantile(0.75) - y_data.quantile(0.25)

# Main scatter plot
ax_scatter.scatter(x_data, y_data, color=colors, s=200, marker='x', linewidths=3)
ax_scatter.set_xlim(0, 1.02)
ax_scatter.set_ylim(0, 1.02)
ax_scatter.set_xlabel("CCF", fontsize=24)
ax_scatter.set_ylabel("blast fraction", fontsize=24)
ax_scatter.grid(color='gray', linestyle='--', linewidth=2, alpha=0.7)
ax_scatter.tick_params(axis='both', width=2, length=6, labelsize=20)
ax_scatter.spines["top"].set_visible(False)
ax_scatter.spines["right"].set_visible(False)
ax_scatter.spines["bottom"].set_linewidth(2)
ax_scatter.spines["left"].set_linewidth(2)

stats_text_x = (
    f"  mean = {mean_x:.2f}\n"
    f"  median = {median_x:.2f}\n"
    f"  IQR = {iqr_x:.2f}\n"
)
plt.text(-4, 1.05, stats_text_x, fontsize=18,
         bbox=dict(facecolor="white", alpha=0, edgecolor="none"))
stats_text_y = (
    f"  mean = {mean_y:.2f}\n"
    f"  median = {median_y:.2f}\n"
    f"  IQR = {iqr_y:.2f}"
)
plt.text(1, 0.85, stats_text_y, fontsize=18,
         bbox=dict(facecolor="white", alpha=0, edgecolor="none"))

# Add correlation text
ax_scatter.text(0.05, 0.85, f"r = {corr:.2f}\np = {p:.2f}",
                fontsize=20, bbox=dict(facecolor="white", alpha=0.6, edgecolor="none"))
# Top boxplot (X-axis)
box_x = ax_histx.boxplot(x_data, vert=False, widths=0.5, patch_artist=False)
for element in ['boxes', 'whiskers', 'caps', 'medians']:
    for item in box_x[element]:
        item.set_linewidth(2.5)  # match scatter plot linewidth
ax_histx.axis("off")

# Right boxplot (Y-axis)
box_y = ax_histy.boxplot(y_data, vert=True, widths=0.5, patch_artist=False)
for element in ['boxes', 'whiskers', 'caps', 'medians']:
    for item in box_y[element]:
        item.set_linewidth(2.5)
ax_histy.axis("off")

# Title
fig.suptitle("CCF vs blast fraction", fontsize=26, x=0.3)

# Save and show
plt.savefig("ccf_blast_with_marginal_boxplots.tif", dpi=200, bbox_inches="tight", facecolor="white")
plt.show()
plt.close()
```
